# MECP2 mutations disrupt pluripotent stem cell fate through remodeling of the three-dimensional genome

**DOI:** 10.1038/s41419-026-08837-4

**Published:** 2026-05-08

**Authors:** Jing Zhou, Yizhuo Che, Xintao Jing, Fang Li, Hang Peng, Yuchun Liu, Li Cao, Jinyuan Zhang, Xiaofei Wang, Jia Zhang, Aihong Guo, Dongdong Tong, Bingju Wang, Chen Huang

**Affiliations:** 1 Department of Cell Biology and Genetics, School of Basic Medical Sciences, Xi’an Jiaotong University, Xi’an, China; 2 Central Laboratory, The Affiliated Children’s Hospital of Nanchang Medical College, Nanchang, China; 3 Faculty of Electronic and Information Engineering, Xi’an Jiaotong University, Xi’an, China; 4 Institute of Basic Medical Sciences, Xi’an Medical University, Xi’an, China; 5 Guangxi Health Science College, Nanning, China; 6 Department of Thoracic Surgery, The First Affiliated Hospital of Xi’an Jiaotong University, Xi’an, China; 7 Department of Neurology, Xianyang Hospital of Yan’an University, Xianyang, China

**Keywords:** Induced pluripotent stem cells, Chromatin remodelling, Disease model, Pluripotency

## Abstract

Mutations in the MECP2 gene are the primary cause of Rett syndrome, yet their mechanistic roles during early developmental stages remain poorly understood. In this study, CRISPR-Cas9 technology was applied to generate three loss-of-function mutations in male induced pluripotent stem cells (iPSCs), namely MECP2^del6^, MECP2^insA^, and MECP2^insT^, each targeting distinct functional domains of MECP2. Our results showed that MECP2 mutations led to reduced proliferative capacity and impaired embryoid body formation in iPSCs, and caused premature loss of OCT4 expression during embryoid body development. To explore the molecular mechanisms in depth, we performed integrated multi-omics analyses. MECP2 mutations remodeled three-dimensional genome organization by disrupting chromatin compartmentalization, destabilizing topologically associated domain boundaries, and redistributing frequent interaction hotspots and super-hotspots linked to genes involved in development and chromatin remodeling. These structural alterations were accompanied by genome-wide changes in chromatin accessibility, with differentially open regions enriched for the binding motifs of pluripotency transcription factors OCT4/SOX2 and the 3D genome organizer CTCF. Further analyses confirmed that the MECP2 mutations enhanced CTCF binding at its co-binding sites. Collectively, this study systematically elucidates how MECP2 mutations interfere with iPSC fate determination by reshaping 3D genome organization and chromatin accessibility at multiple levels, providing a new perspective on the early pathogenesis of Rett syndrome.

## Introduction

The Methyl-CpG binding protein 2 (MECP2) gene, located on the X chromosome, is a critical epigenetic regulator that modulates gene expression and chromatin structure by recognizing DNA methylation marks [1]. Mutations in MECP2 are the primary cause of Rett syndrome (RTT), a severe neurodevelopmental disorder that predominantly affects females due to X-linked inheritance [2–4]. RTT is characterized by a period of apparently normal development for the first 6–18 months, followed by a progressive loss of motor and language skills, stereotypic hand movements, and other hallmark neurological impairments [5, 6]. In addition to these core features, RTT patients often present with multi-system abnormalities, including respiratory irregularities, gastrointestinal dysfunction, and skeletal deformities, highlighting the essential role of MECP2 in the development and function of multiple organ systems [7, 8].

Despite significant advances in understanding MECP2 function in neurons, its specific role during early developmental stages and in pluripotent stem cells remains poorly understood. This knowledge gap is partly due to limitations of current research models: MECP2 mutations are lethal in male embryos, making patient-derived stem cell models difficult to obtain [9]. While in female cells, random X chromosome inactivation creates mosaic expression that adds further complexity [10]. The X chromosome inactivation status has been shown to change dynamically during reprogramming, and MECP2 mutations may induce aberrant reactivation of genes on the previously inactive X chromosome [11]. Although MECP2 is recognized as a key regulator of early embryonic development, it does not appear to markedly affect basic cell growth at the stem cell stage [12]. Although previous studies have reported MECP2 mutation engineering in human iPSCs and chromatin association defects in differentiated neurons [13, 14]. The role of MECP2 in shaping three-dimensional genome architecture in human pluripotent stem cells has not been systematically characterized. Thus, our study addresses a distinct regulatory layer and developmental stage by integrating multi-omics approaches to define how MECP2 dysfunction reorganizes 3D genome architecture and impacts pluripotent cell fate. These limitations constrain our ability to systematically resolve the early pathogenic mechanisms of Rett syndrome and to develop targeted interventions. Therefore, investigating the regulatory functions of MECP2 at the chromatin architecture level in iPSC models is critical for elucidating the developmental origins of RTT.

In recent years, studies have revealed that MECP2 is not only a reader of DNA methylation but also a key regulator of higher-order chromatin structure, participating in its formation and dynamic regulation at multiple levels. MECP2 can directly bind nucleosomes and compete with linker histone H1 for binding sites, thereby compressing nucleosome arrays and promoting higher-order chromatin folding and condensation [15]. MECP2 is enriched in heterochromatin and contributes to its compartmentalization by interacting with factors such as HP1 proteins and major satellite RNAs, thereby preserving its spatial structure and functional integrity [16, 17]. Beyond its classical roles in DNA binding and chromatin compaction, MECP2 also contains intrinsically disordered regions (IDRs) that enable liquid–liquid phase separation, facilitating the formation of biomolecular condensates. This property offers a new mechanistic perspective on how MECP2 dynamically regulates chromatin spatial organization [18]. Moreover, MECP2 interacts with structural proteins such as cohesin and ATRX, contributing to chromatin loop formation and the maintenance of three-dimensional genome architecture [19]. Collectively, these findings establish MECP2 as a multifunctional regulator that links epigenetic information to higher-order chromatin organization. However, how aberrant MECP2 expression disrupts 3D genome structure and leads to functional consequences remains an important unresolved question in the field.

In this study, we investigated the role of MECP2 in shaping the three-dimensional genomic and chromatin landscapes of human induced pluripotent stem cells using a multi-omics approach. We generated three homozygous iPSC lines carrying distinct loss-of-function mutations in key functional domains of MECP2. These mutant lines displayed impaired phenotypes, including slowed proliferation and reduced clone formation efficiency, with severity correlating to the extent of protein truncation. MECP2 defects disrupted embryoid body formation, manifesting as morphological abnormalities, diminished cavity development, and early down-regulation of the pluripotency marker OCT4, suggesting impaired differentiation capacity. To elucidate the molecular mechanisms underlying these phenotypic alterations, we conducted integrative analyses of 3D genome organization, chromatin accessibility, and gene expression. High-resolution Hi-C mapping showed that MECP2 mutations induce widespread changes in chromatin architecture, reflected in global alterations of compartmental states, pronounced shifts in TAD boundary organization, and modified insulation strength at defined genomic regions. 3C experimental validation confirmed that these structural changes are functionally associated with aberrant promoter–enhancer interactions and consequent gene dysregulation. Furthermore, we identified specific alterations in frequently interacting regions and their superclusters. Genes located within the altered superclusters were significantly enriched for components of chromatin remodeling complexes, while control-specific FIREs were predominantly linked to developmental processes. At the same time, ATAC-seq analysis revealed widespread, mutation-specific alterations in chromatin accessibility across the genome. Motif analysis of differentially accessible regions demonstrated significant enrichment of binding sites for pluripotency transcription factors and the architectural protein CTCF. These findings prompted a detailed investigation of CTCF occupancy. ChIP-seq profiling showed a genome-wide increase in CTCF binding strength at sites that normally co-bind with MECP2 in wild-type cells. Subsequent 3 C assays at these loci provided direct evidence that aberrant CTCF binding is associated with altered local chromatin looping and dysregulated gene expression.

In summary, our findings underscore the multifaceted role of MECP2 in safeguarding the epigenetic landscape of iPSCs. We show that MECP2 deficiency drives coordinated disruptions across several layers of genomic regulation, including both large-scale and local chromatin organization, chromatin accessibility, and transcription factor binding. These interconnected alterations, particularly those involving CTCF-mediated genome organization, provide a mechanistic basis for understanding how MECP2 mutations perturb the transcriptional networks that sustain pluripotency and direct early cell fate decisions.

## Results

### Mutations in the *MECP2* gene impair cell growth in iPSCs

The *MECP2* gene is located on the long arm of the X chromosome at Xq28 and spans approximately 76 kb. It comprises four exons and three introns, which undergo alternative splicing to generate two major transcript isoforms: *MECP2*-E1 and *MECP2*-E2. The MECP2 protein consists of five major structural domains: the N-terminal domain (NTD), the methyl-binding domain (MBD), the inter-domain (ID), the transcriptional repression domain (TRD), and the C-terminal domain (CTD).

Given that the MECP2 gene is located on the X chromosome and is X-linked, the expression pattern of iPSCs originating from females carrying the MECP2 mutation is not well distinguished from normal iPSCs [11]. Cellular phenotypic heterogeneity triggered by random X chromosome inactivation (XCI) can be effectively avoided using male iPSCs carrying mutations in the MECP2 gene. However, loss-of-function mutations in the MECP2 gene lead to a lethal phenotype in male embryos during development, and clinically reported cases of surviving males carrying MECP2 mutations are extremely rare. For these reasons, in the present study, we chose to obtain dermal fibroblasts from healthy adult male volunteers and transform them into iPSCs by reprogramming and editing MECP2 by CRISPR as the experimental materials [20].

**Fig. 1:**
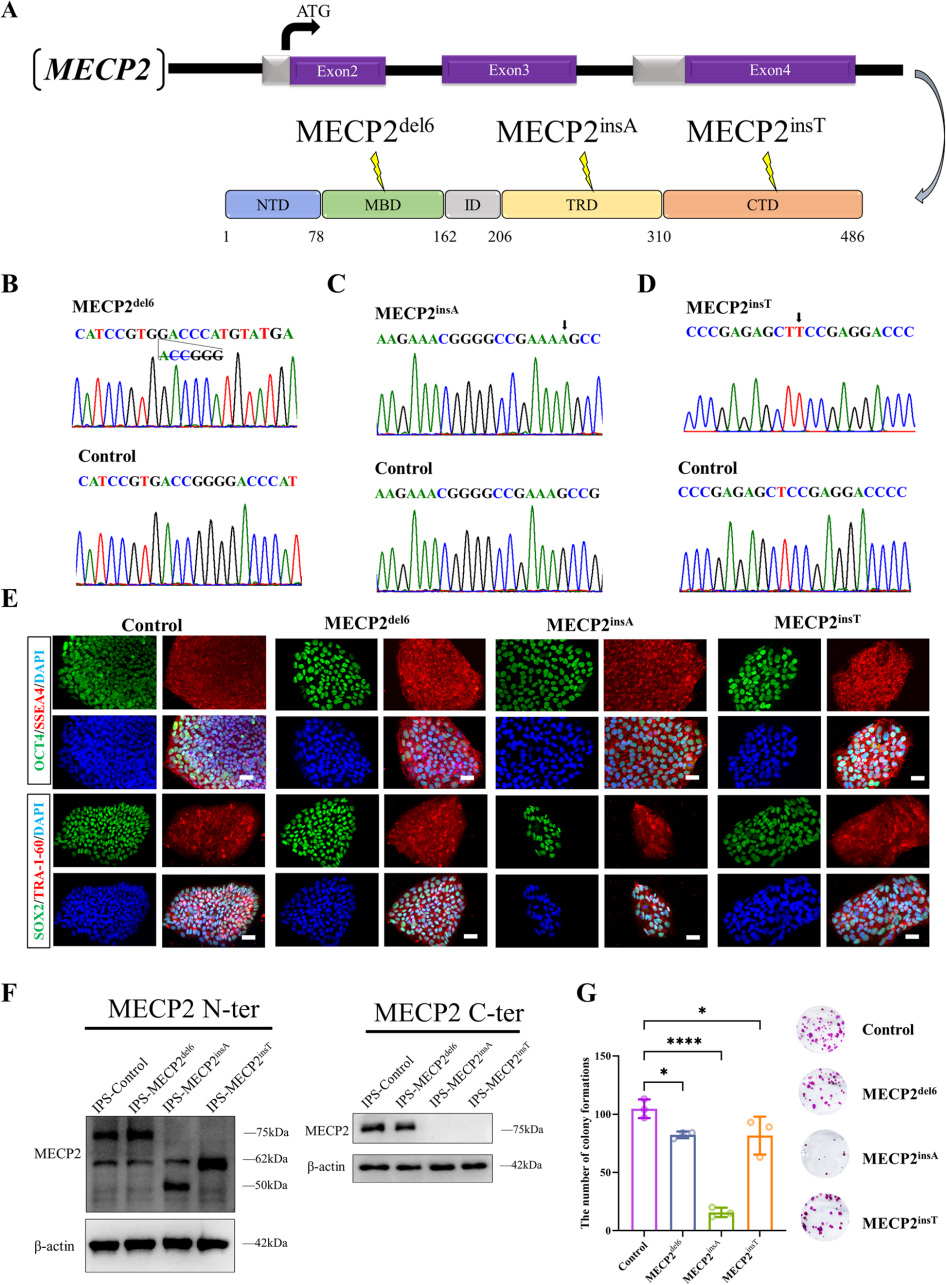
Generation of male iPSCs carrying three distinct MECP2 mutations from normal control iPSCs. **A** Structure of the MECP2 gene and CRISPR target sites. Three pairs of CRISPR gRNAs were designed to target the MBD, TRD, and CTD domains, generating representative MECP2 mutation types. **B** MECP2^del6^: an MBD domain deletion mutation (NM_004992: exon3: c.269_275del, p.D90_R91del). **C** MECP2^insA^: a TRD domain frameshift mutation (NM_004992: exon4: c.812insA p. P271AfsTer58). **D** MECP2^insT^: a CTD domain frameshift mutation (NM_004992: exon4: c.1186insT, p. S396FfsTer7). **E** Immunofluorescence staining of pluripotency markers OCT4, SOX2, SSEA4, TRA-1-60 in control and mutant lines. All groups showed positive expression. Scale bar = 50 μm. **F** Western blot analysis of MeCP2 protein expression with N-terminal-specific MECP2 antibody and C-terminal-specific MECP2 antibody. **G** Colony formation assay of control iPSCs versus MECP2^del6^, MECP2^insA^, and MECP2^insT^ iPSCs. All mutant groups formed fewer colonies compared to controls. *n* = 3 biological replicates. Data are presented as means ± SD, unpaired Student’s *t* test. *p < 0.05, **p < 0.01; ***p < 0.001; ****p < 0.0001.

Due to the essential role of *MECP2*, the genotypes of mutant iPSCs capable of surviving the pluripotent stage remain largely unknown. In this study, we employed CRISPR-Cas9 to target the MBD, TRD, and CTD of MECP2, which represent major mutational hotspots in RTT [21] (Fig. 1A). Previous genetic studies have shown that pathogenic MECP2 mutations are highly enriched in the MBD and TRD regions, while truncating mutations affecting the C-terminal region are also frequently observed in RTT patients and collectively account for the majority of reported cases [22]. Based on this mutational landscape, we generated three distinct MECP2 mutant iPSC lines representing different classes of RTT-associated mutations: MECP2^del6^, carrying a 6-bp deletion within the MBD region (Fig. 1B); MECP2^insA^, containing a single-base (A) insertion within the TRD region that results in a frameshift and premature protein truncation (Fig. 1C); and MECP2^insT^, harboring a single-base (T) insertion in the CTD region leading to C-terminal truncation (Fig. 1D). Together, these mutations model three major categories of MECP2 alterations observed in RTT, including MBD-associated mutations, TRD-associated truncating mutations, and C-terminal deletions. Karyotype analysis confirmed that all three mutant lines retained a normal male karyotype (46, XY), with no detectable chromosomal abnormalities introduced during CRISPR editing (Fig. S1A–D). To assess whether gene editing affected pluripotency, we performed immunofluorescence staining for the pluripotency markers SOX2, OCT4, TRA-1-60, and SSEA4. All three mutant lines exhibited marker expression comparable to the wild-type control, indicating preserved stem cell identity (Fig. 1E). Because gene editing introduced base deletions and frameshift mutations in MECP2, the effects on MECP2 protein stability and integrity were initially unclear. To assess this, we first performed Western blot analysis using a C-terminal–specific MECP2 antibody. MECP2^del6^ exhibited detectable MECP2 protein with molecular weight and expression levels comparable to those of the control. In contrast, MECP2^insA^ (329 aa) and MECP2^insT^ (403 aa) were not detected, consistent with premature termination caused by frameshift mutations. We then performed WB analysis using an N-terminal–specific MECP2 antibody. Truncated proteins corresponding to the predicted sizes of MECP2^insA^ and MECP2^insT^ were detected, and their expression levels did not differ significantly from controls (Fig. 1F and S1E, F).

We next assessed the growth characteristics of the mutant iPSC lines. MECP2^insA^ and MECP2^insT^ showed reduced proliferation rates compared with the wild-type control, whereas MECP2^del6^ did not display a significant difference (Fig. S1G). Notably, the degree of growth impairment correlated positively with the extent of MECP2 protein truncation: MECP2^insA^ showed the most pronounced growth defect, followed by MECP2^insT^, while MECP2^del6^ displayed only a modest reduction in growth relative to the control. Similarly, colony formation assays revealed significantly lower cloning efficiency in all three mutant lines compared to the control (Fig. 1G), again showing a trend consistent with the severity of MECP2 disruption. These findings indicate that *MECP2* mutations impair the growth and clonogenic capacity of iPSCs, suggesting that MECP2 plays an essential role in maintaining stem cell viability and proliferative potential at the pluripotent stage.

### MECP2 mutations alter the morphology of embryoid bodies

**Fig. 2:**
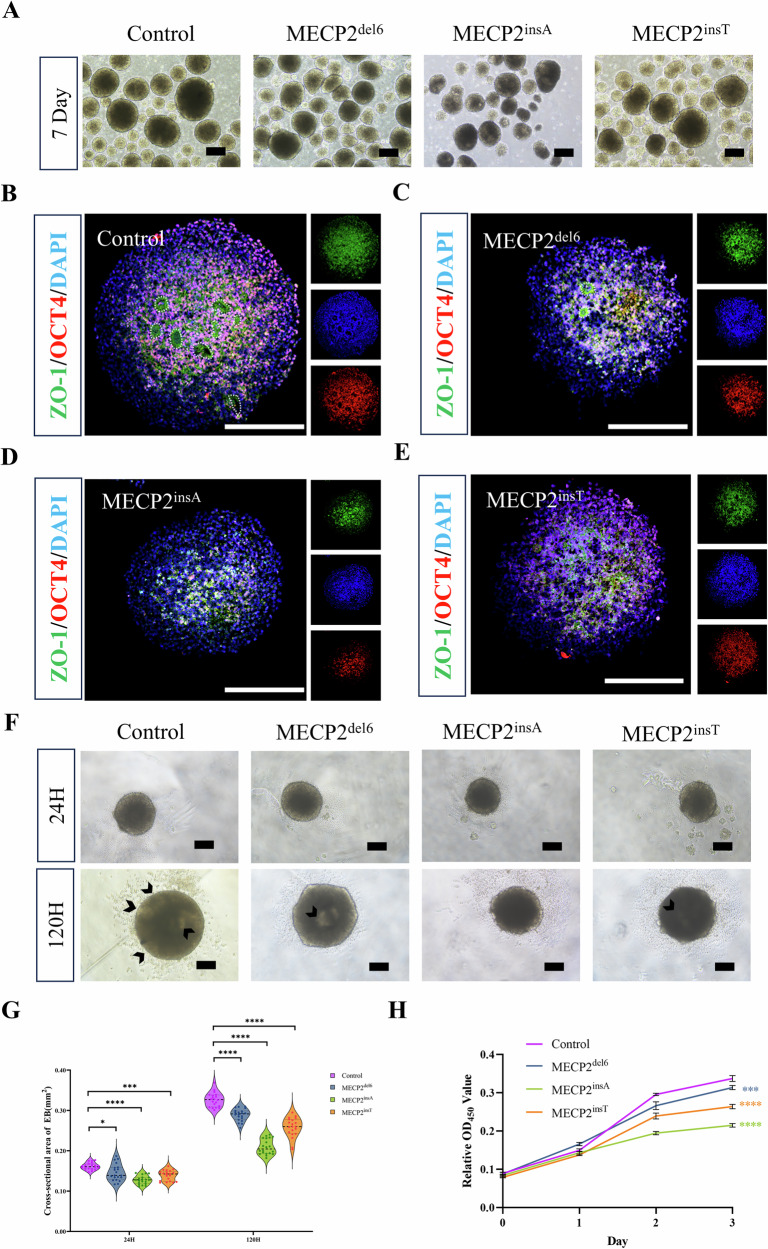
MECP2 mutations impair embryoid body growth and morphology. **A** Control and mutant EBs after 7 days of differentiation in Essential 6 medium. **B**–**E** Immunofluorescence of frozen EB sections from control and mutant iPSCs following 7-day spontaneous differentiation in Essential 6 medium. ZO-1 (green), OCT4 (red), and DAPI (blue) staining are shown. White dashed circles highlight hollow structures, which were reduced or absent in *MECP2* mutants. Scale bar = 50 µm. **F** Spontaneous differentiation assay in U-bottom 96-well plates seeded with 10,000 cells/well. Black arrows indicate hollow structures. Scale bar = 200 µm. **G** Growth trajectory analysis of EBs from 24 to 120 h, comparing control and MECP2^del6^, MECP2^insA^, and MECP2^insT^ groups. Statistically significant growth differences emerged from 24 h onward. At least 20 EBs were analyzed in each group. **H** Proliferation curves of control and MECP2 mutant iPSCs in Essential 6 medium. All mutant groups exhibited reduced growth rates compared to controls. *n* = 3 biological replicates. Data are presented as means ± SD, unpaired Student’s *t* test. *p < 0.05, **p < 0.01; ***p < 0.001; ****p < 0.0001.

To evaluate the tri-lineage differentiation potential of MECP2 mutant iPSCs compared to control iPSCs, we induced spontaneous differentiation by generating EBs and culturing them in suspension using Essential 6 medium for 7 days (Fig. 2A). All three mutant lines successfully formed EBs, which were subsequently plated and cultured for an additional 3 days under adherent conditions. Immunofluorescence staining confirmed that both mutant and control iPSCs expressed lineage-specific markers corresponding to the three embryonic germ layers ectoderm, mesoderm, and endoderm indicating that MECP2 mutations did not compromise the core pluripotent differentiation capacity (Fig. S2A–D). Despite similar marker expression, we observed notable morphological differences between EBs derived from *MECP2* mutants and those from the control group during the course of differentiation (Fig. 2A). Cryosectioning and immunofluorescence staining of embedded EBs revealed the presence of internal cavity structures in the control group, which were largely absent or disrupted in the mutants (Fig. 2B). These observations suggest that while *MECP2* mutant iPSCs retain the ability to differentiate into all three germ layers, the mutations impair proper EB morphogenesis. During EB formation, cavity structures typically emerge as a result of coordinated cell aggregation and early differentiation processes. These cavities resemble the yolk sac cavity observed during the blastocyst stage and represent a key morphological feature of early embryonic development. Moreover, proper cavity formation is closely associated with the differentiation potential of embryonic stem cells [23]. In our study, all three *MECP2* mutant lines exhibited a marked reduction in the number of cavity structures, which were either significantly diminished or nearly undetectable (Fig. 2C–E). In parallel, we observed decreased expression of the pluripotency marker OCT4 in all three mutant groups compared to the control, as demonstrated by immunofluorescence staining (Fig. 2B–E). To further strengthen this observation, we performed time-course qPCR analysis of *OCT4* during EB differentiation and found a significant reduction in OCT4 expression in all MECP2 mutant EBs relative to control from day 6 onward (Fig. S2E). Together, these findings suggest that MECP2 mutations impair not only EB morphogenesis but also the maintenance of pluripotency during early differentiation.

To assess the spontaneous differentiation potential, we employed the clonal clump formation method as previously described [24]. For quantifying EB formation and growth, iPSCs were enzymatically dissociated into single cells and seeded into U-bottom 96-well plates. Gentle centrifugation was applied to facilitate cell aggregation while minimizing variability (Fig. S2F). The resulting EBs displayed morphology comparable to that generated using traditional methods. By day 5, clear differences in cavity structure were observed between the control and the three MECP2 mutant groups (Fig. 2F). Specifically, EBs in the control group developed more and larger internal cavities, whereas those in the mutant groups exhibited smaller and fewer cavities. Moreover, the growth rate of EBs was significantly reduced in MECP2 mutant lines compared to controls and showed a positive correlation with the severity of MECP2 dysfunction, mirroring the trend observed at the iPSC stage (Fig. 2G). To further validate these findings, we assessed cell growth under 2D adherent conditions. Consistent with the 3D EB results, MECP2-mutant iPSCs also exhibited impaired growth in 2D culture (Fig. 2H). Collectively, these results indicate that MECP2 mutations impair the spontaneous differentiation capacity of iPSCs.

### MECP2 mutations affect the 3D genomic structure of iPSCs

To investigate the global 3D genome folding patterns following MECP2 mutations, we performed Hi-C analysis on two mutant models, MECP2^insA^ and MECP2^del6^. We first examined the distance-dependent decay of chromatin interaction frequency. Contact probability decay curves were fitted using a power-law model. MECP2^insA^ and MECP2^del6^ exhibited increased contact probabilities at short genomic distances, together with more negative scaling exponents compared with control, indicating a steeper distance-dependent decay of chromatin interactions (Fig. S3A). Next, we analyzed chromatin compartmentalization by partitioning the genome into active A and inactive B compartments. Both MECP2^insA^ and MECP2^del6^ displayed an increased proportion of active A compartments relative to controls (Fig. S3B). Notably, MECP2 mutations induced extensive compartment switching. In MECP2^insA^, 21.73% of B compartments transitioned to A compartments, while 26.00% of A compartments transitioned to B compartments. Only 52.27% of compartments remained unchanged, including 20.11% A compartments and 32.16% B compartments (Fig. S3C). Similar patterns were observed in MECP2^del6^, where only 56.45% of compartments remained stable, comprising 25.15% A compartments and 31.30% B compartments (Fig. S3D). These results indicate highly dynamic changes in chromatin contacts and compartment activity following MECP2 mutation (Fig. S3E, F).

**Fig. 3:**
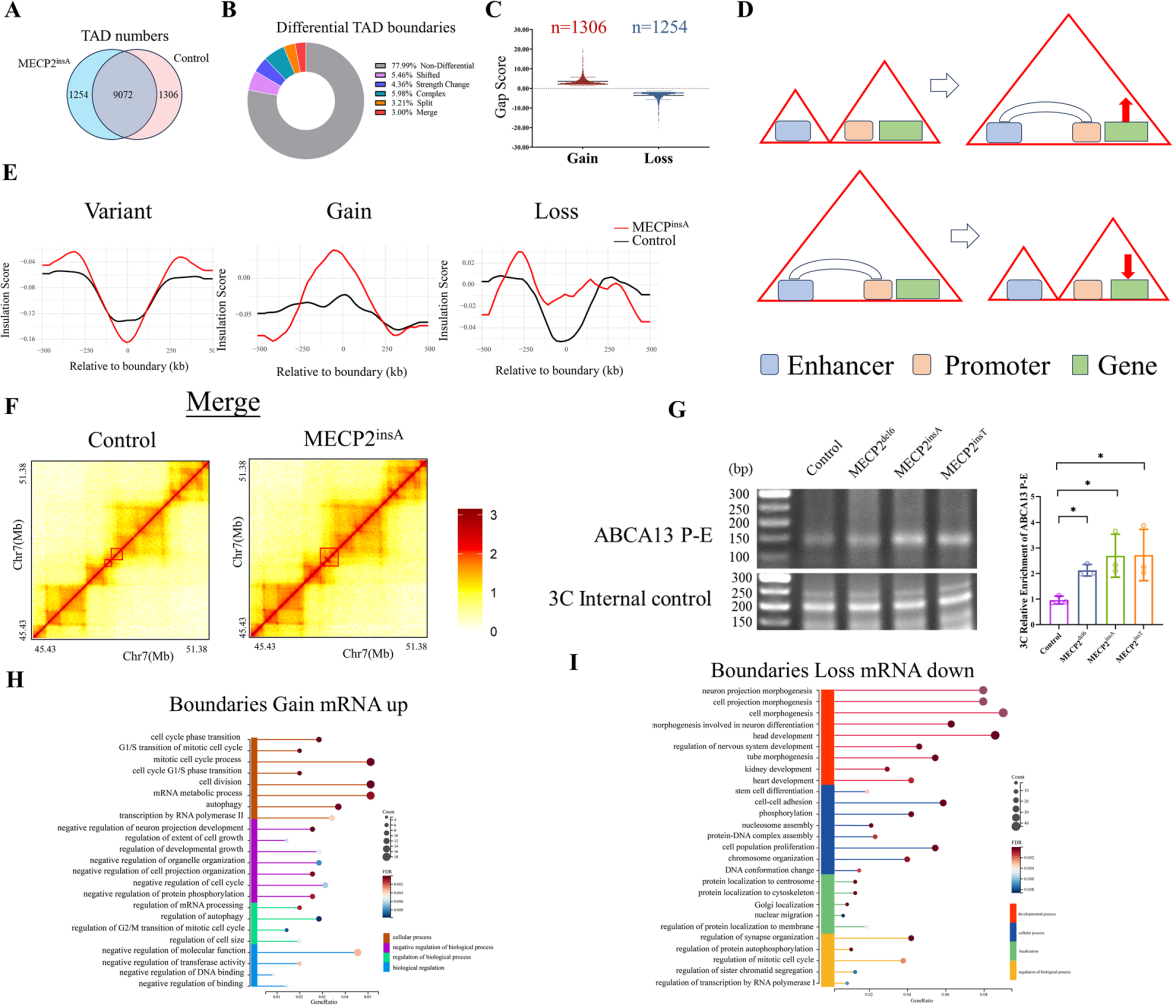
MECP2 mutations drive TAD boundary reorganization and functional genomic alterations. **A** TAD calling at 10-kb resolution identified 10,326 in MECP2^insA^ and 10,378 in Control TADs, with 9072 shared between groups. **B** Classification of TAD boundary dynamics: 77.99% unchanged; 5.46% shifted; 4.36% strength-changed; 5.96% complex; 3.21% split; 3.00% merged. **C** Differential boundary analysis: 1306 gained and 1254 lost boundaries in MECP2^insA^ versus Control. **D** Schematic illustration of potential effects of TAD boundary merging or splitting on enhancer–promoter interactions. **E** Average insulation score profiles for invariant, gained, and lost TAD boundaries in control and MECP2^insA^ iPSCs. **F** Hi-C contact map showing a representative TAD merge region on chromosome 7 encompassing the ABCA13 locus. **G** 3C analysis of promoter–enhancer interactions at the ABCA13 locus in control and MECP2 mutant iPSCs. Expression statistics results are derived from RNA-seq. *n* = 3. Data are presented as means ± SD, unpaired Student’s *t* test. **p < 0.01; ***p < 0.001; ****p < 0.0001. **H** GO enrichment analysis of genes located near gained TAD boundaries with increased expression (Gain + UP). **I** GO enrichment analysis of genes located near lost TAD boundaries with decreased expression (Loss + Down).

Given that MECP2^insA^ and MECP2^del6^ exhibited consistent alterations across multiple fundamental Hi-C features, we selected MECP2^insA^ as a representative model for subsequent in-depth analyses of higher-order three-dimensional chromatin structures. We therefore sought to identify genomic regions where TAD rearrangements occur following MECP2 mutations. At a resolution of 10 kb, we detected 10,326 and 10,378 TADs in the MECP2^insA^ and control samples, respectively (Fig. 3A), with 9072 TADs shared between both groups. Analysis of TAD boundary dynamics revealed that 77.99% of boundaries were classified as invariant. The remaining boundaries exhibited varying degrees of structural changes: 5.46% were shifted, 4.36% showed changes in insulation strength, 5.96% were categorized as complex, 3.21% were split, and 3.00% were merged (Figs. 3B, F and S4A–D, Supplementary Table 1). These levels of TAD boundary reorganization indicate substantial chromatin architecture remodeling in MECP2^insA^, and appear more extensive compared to previously reported datasets [25, 26]. We classified the identified TAD-variant boundaries into two categories: gained and lost. Gained boundaries (*n* = 1306) refer to those present only in MECP2^insA^, while lost boundaries (*n* = 1254) were present in controls but absent in MECP2^insA^ (Fig. 3C). Changes at the boundaries of TADs can influence gene expression. For example, the merging of TAD edges can create new TADs in which enhancer-promoter contacts are strengthened, resulting in increased gene expression. Conversely, TAD splitting may reduce enhancer-promoter interactions, leading to decreased gene expression (Fig. 3D). To assess boundary strength, we analyzed the average insulation scores for gained, lost, and invariant boundaries. As expected, insulation scores at invariant boundaries were comparable between MECP2^insA^ and control samples. In contrast, gained boundaries showed significantly higher insulation scores in MECP2^insA^ compared to controls, while lost boundaries exhibited significantly reduced insulation in MECP2^insA^ (Fig. 3E). These results support the robustness of our TAD boundary classification and validate the reliability of the identified boundary changes. To validate the reliability of our findings, we performed 3C analysis to assess contact strength within representative signature regions (Fig. S4E). We selected a TAD merge region on *Chr7:45,434,228–51,384,727* (Fig. 3F), which encompasses ABCA13, a gene previously implicated in schizophrenia and autism spectrum disorders [27]. RNA-seq analysis was conducted for all three MECP2 mutant lines (Supplementary Table 2). Using 3C, we examined promoter–enhancer interactions at the ABCA13 locus by targeting the promoter and inferred enhancer regions located upstream and downstream of the TAD merge site across all three mutants (Fig. 3G). Following the MECP2 mutation, we observed enhanced promoter–enhancer contact strength accompanied by increased ABCA13 expression in all mutant lines (Fig. S4F). We assigned genes to regions adjacent to gained or lost TAD boundaries and integrated these data with transcriptomic analyses (Supplementary Table 1). The results showed that genes located near lost TAD boundaries did not exhibit significant differences in overall enrichment compared with those near newly gained TAD boundaries when comparing MECP2^insA^ and control groups (Fig. S4G). Previous studies have suggested that transcriptional dysregulation resulting from TAD structural alterations may become apparent only within specific developmental time windows [28]. Therefore, the lack of pronounced differences observed here may be attributable to the generally low transcriptional activity of most genes at the iPSC stage. Next, we focused on genes affected by TAD boundary alterations, as these represent candidate targets that are particularly sensitive to TAD boundary rearrangements during the iPSC stage and exhibit corresponding transcriptional responses. To establish a more direct association between TAD boundary changes and gene regulation, we further restricted our analysis to genes that were both located near regions of TAD boundary gain or loss and showed significant expression changes in the RNA-seq data (Supplementary Table 1). Based on the type of boundary alteration and the direction of expression change, these genes were classified into two groups: genes located near newly gained TAD boundaries with increased expression (Gain + UP) and genes located near lost TAD boundaries with decreased expression (Loss + Down). GO enrichment analysis of the Gain + UP genes revealed significant enrichment in parent categories associated with cell cycle-related cellular processes (Fig. 3H). This is consistent with previous findings showing that MECP2 deficiency during the reprogramming of fibroblasts into iPSCs leads to premature activation of G1–S phase transitions, abnormal spindle geometry, and prolonged mitosis [29, 30]. Beyond neurodevelopmental effects, individuals with RTT frequently exhibit peripheral symptoms affecting multiple organ systems, including the cardiac, pulmonary, muscular, skeletal, gastrointestinal, and immune systems. These systemic manifestations have been validated in several animal models, including mouse and Cynomolgus monkey [31, 32], aligning with the broad developmental pathways identified in our GO enrichment analysis, particularly those associated with the negative regulation of biological processes. Additionally, our GO results indicate significant enrichment in parent categories related to negatively regulated pathways within broader biological regulation terms. Correspondingly, GO enrichment analysis of the Loss + Down genes showed significant enrichment in developmental pathways, including nervous system development and cell projection organization (Fig. 3I), suggesting that lost TAD boundaries are closely associated with the regulation of neural development. GO enrichment analysis also revealed significant enrichment in parent categories related to cellular processes, including phosphorylation, protein–DNA complex assembly, and chromosome organization. In mouse cells, MECP2 has been shown to accumulate at chromocenters and co-localize with ATRX in these heterochromatic regions, where it plays a role in regulating gene expression in terminally differentiated neurons [33]. Consistent with these findings, our enrichment analysis also highlighted GO terms related to subcellular localization processes, including protein localization to the centrosome and nuclear migration, further supporting the role of MECP2 in maintaining nuclear architecture and chromatin organization. In summary, our data support a model in which MECP2 influences higher-order chromatin architecture and cell fate decisions by altering chromatin compartmental activity, inducing TAD boundary rearrangements, and regulating the spatial organization of developmentally relevant genes within the 3D genome of iPSCs.

### MECP2-dependent FIREs/Super-FIREs are associated with cell fate-specific gene regulation

**Fig. 4:**
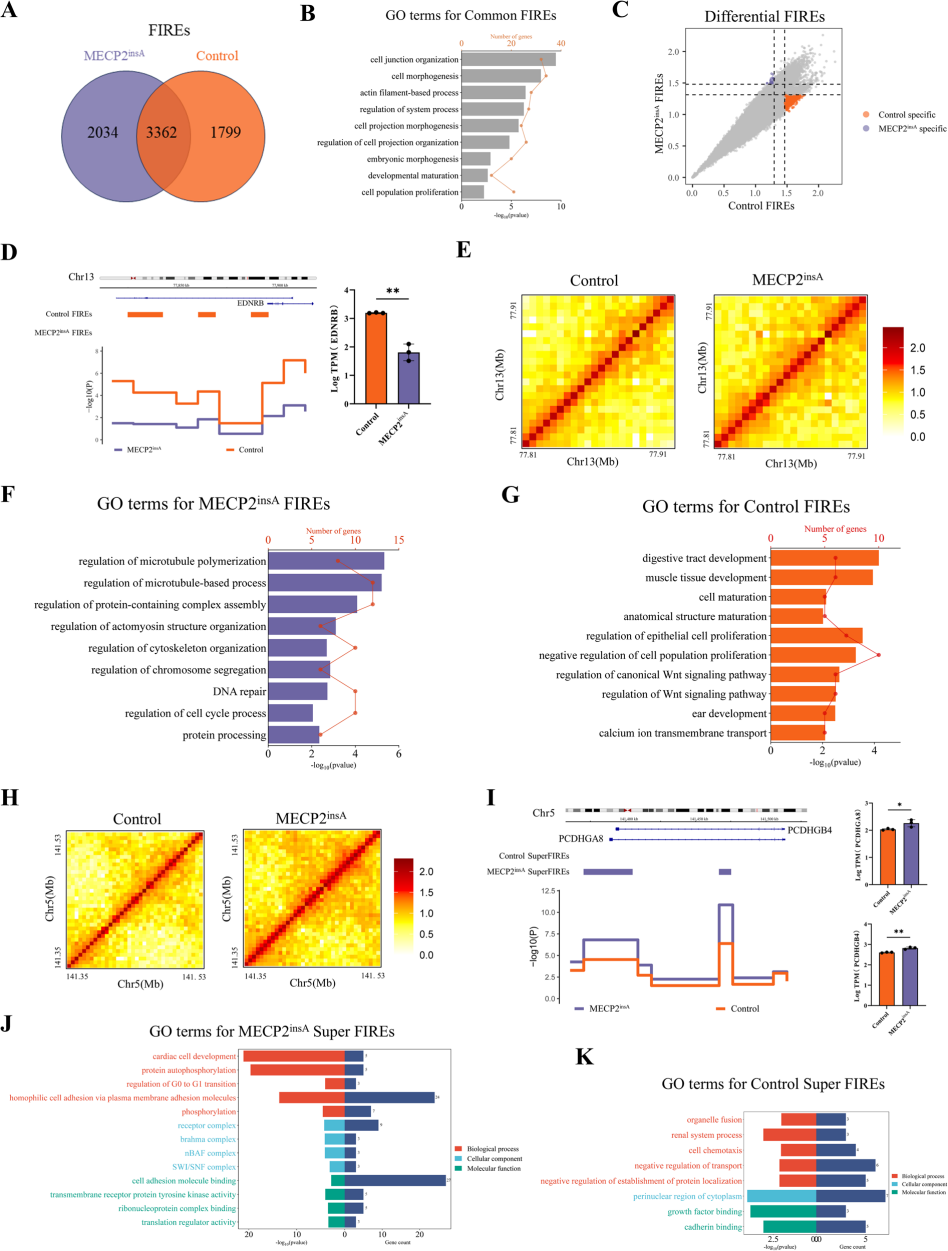
MECP2 mutations disrupt local chromatin interactions in FIREs and super-FIREs. **A** Venn diagram shows the number of FIREs identified in control and MECP2^insA^ iPSCs, along with the number of FIREs shared between the two groups. **B** GO biological process terms enriched for genes associated with common FIREs. **C** Four-quadrant volcano plot showing the number of MECP^*2insA-*^specific and control-specific FIREs, as well as the overlapping FIREs between the two groups. **D** Genome browser tracks showcasing representative FIRE signals at the *EDNRB* gene locus in Control and MECP2^insA^ iPSCs. RNA-seq TPM values indicate that *EDNRB* expression is reduced in MECP2^insA^ iPSCs. **E** Hi-C contact heatmap of the *EDNRB* gene locus. **F**, **G** GO BP analysis of genes associated with MECP2^insA^-specific FIREs and Control-specific FIREs. **H** Hi-C contact heatmap of MECP2^insA^ representative super-FIRE locus: *chr5:141,353,037*−*141,533,551*. **I** Genome browser tracks showcasing representative FIRE signals at *chr5:141,353,037*−*141,533,551* in Control and MECP2^insA^ iPSCs. RNA-seq analysis of *PCDHGA8* and *PCDHGB4* within the relevant FIRE/Super-FIRE regions revealed increased expression levels in MECP2^insA^ iPSCs. **J** GO analysis of genes within MECP2^insA^-specific Super-FIREs. **K** GO analysis of genes within control-specific Super-FIREs.

Frequent interaction regions (FIREs) are cell-type-specific local interaction hotspots that contain active cis-regulatory elements [34]. Contiguous FIREs can form clusters known as Super-FIREs, which exhibit the highest local interaction frequencies. To investigate whether MECP2 influences FIREs and Super-FIREs in iPSCs, we identified 5394 and 5161 FIREs in MECP2-mutant and control iPSCs (Fig. 4A). Using coordinate-level overlap and similarity scores [35]. We identified 721 Common FIREs shared between the two samples, corresponding to 585 genes (Supplementary Table 1). These genes were predominantly associated with housekeeping functions, including cell morphogenesis, actin filament-based processes, regulation of cell projection organization, and stem cell-related activities (Fig. 4B). To further investigate the impact of MECP2 on FIREs, we identified 56 MECP2^insA^-specific FIREs and 555 control-specific FIREs based on FIRE scoring criteria [25] (Fig. 4C and Supplementary Table 1). FIREs are typically considered enhancer regions, with most exhibiting interactions within their local chromatin neighborhoods [36]. Mapping these specific FIRE regions revealed that the majority are located within the genome, in exons, introns, and coding sequences (CDS), with only a small fraction in promoter and UTR regions (Fig. S5A, B), which is consistent with previous reports [25, 35, 37]. Next, we examined representative regions of the identified FIREs (Figs. 4D, E and S5D), including *EDNRB*, which is regulated by control FIREs and is essential for neural crest stem cell development [38]. In MECP2^insA^ iPSCs, the loss of this FIRE was associated with reduced *EDNRB* expression. We next clustered the genes located within MECP2^insA^-specific FIREs and found that they were enriched for functions related to cytoskeletal dynamics, cell cycle regulation, maintenance of DNA integrity, and protein homeostasis (Fig. 4F). In contrast, genes located within control-specific FIREs were primarily associated with processes related to differentiation and development (Fig. 4G).

Super-FIREs represent a small subset of FIRE clusters that exhibit the highest local interaction frequencies. They are considered to have strong gene regulatory potential and frequently overlap with super-enhancer regions [39]. In our study, we identified 272 Super-FIREs in MECP2^insA^ iPSCs and 241 Super-FIREs in control iPSCs. After removing Super-FIREs common to both genotypes, we obtained 199 MECP2^insA^-specific Super-FIREs and 168 control-specific Super-FIREs (Fig. S5C and Supplementary Table 1). Similar to FIRE regions, Super-FIRE regions exhibit stronger chromatin contacts (Figs. 4H and S5D, E) and correspondingly higher expression of genes within their influence (Fig. 4I). Analysis of genes within Super-FIRE regions revealed that MECP2^insA^-specific Super-FIRE genes were significantly enriched in protein complexes, including the brahma complex, nBAF complex, and SWI/SNF superfamily-type complex (Fig. 4J). This finding is particularly interesting, as the SWI/SNF complex is known to utilize ATP hydrolysis to reposition or evict nucleosomes and thereby promote chromatin accessibility, while MECP2 has also been shown to bind directly to nucleosomes and influence chromatin accessibility [40, 41]. In contrast, genes within control-specific Super-FIREs are involved in processes related to cellular homeostasis, adhesion, and migration (Fig. 4K), including organelle fusion, cell chemotaxis, and cadherin binding. These results suggest that, under normal physiological conditions, one of the primary functions of three-dimensional genomic structures, particularly FIREs, is to maintain and regulate fundamental cellular structure and function.

Overall, MECP2 is essential for preserving the fidelity of the three-dimensional genome in iPSCs, particularly through maintaining the integrity of FIREs and super-FIREs. Its mutation disrupts local chromatin interactions, which consequently alter the expression of key genes that govern cell fate and function.

### MECP2 mutations regulate downstream gene expression by altering chromatin accessibility of iPSCs

**Fig. 5:**
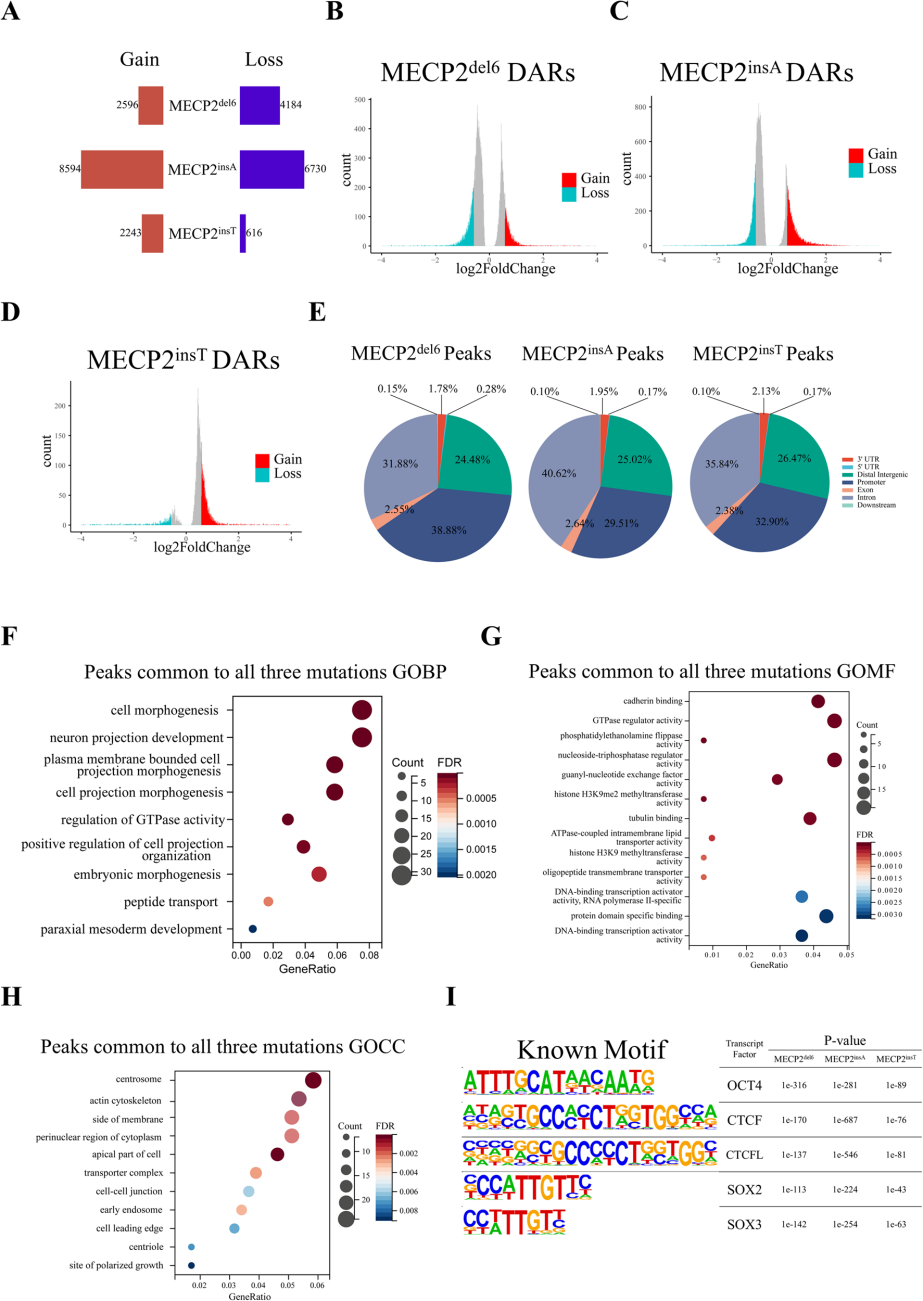
Impact of MECP2 mutations on chromatin accessibility and cellular functions in iPSCs. **A** Comparative analysis of DARs across three MECP2 mutants MECP2^del6^, MECP2^insA^, and MECP2^insT^. **B**−**D** Mutation-specific DAR distributions: MECP2^del6^: 2596 increased/4184 decreased; MECP2^insA^: 8594 increased/6730 decreased; MECP2^insT^: 2243 increased/616 decreased. **E** Genomic annotation of DARs across functional elements. Half of the DARs were distributed in Promoter and Distal intergenic regions, which play a role in the regulation of gene expression. **F** GO biological process enrichment of genes associated with common DARs. **G** GO molecular function analysis of common DAR-associated genes, showing enrichment for: Histone modification, GTPase regulation, and Cell adhesion molecules. **H** GO cellular component analysis revealed DAR-related genes were predominantly localized to the nuclear periphery and cell junctions. **I** Transcription factor motif enrichment analysis of DARs performed using HOMER, showing significant enrichment of pluripotency-associated factors (OCT4/SOX family) and chromatin organizers (CTCF/CTCFL). Statistical significance (*p* values) for the top-enriched motifs is indicated in the figure.

To explore whether the observed changes in chromatin spatial conformation influence the local chromatin environment, we next assessed chromatin accessibility using ATAC-seq. In MECP2^del6^, we identified 2596 differentially accessible regions (DARs) with increased accessibility and 4184 DARs with decreased accessibility (*p*adj < 0.05, |Log₂FC| > 0.58) (Fig. 5A, B and Supplementary Table 3). In MECP2^insA^, there were 8594 DARs with increased accessibility and 6730 with decreased accessibility (Fig. 5A, C and Supplementary Table 3). In MECP2^insT^, we detected 2243 DARs of increased and 616 DARs of decreased chromatin accessibility (Fig. 5A, D and Supplementary Table 3). These results indicate that MECP2 mutations lead to widespread and mutation-specific alterations in chromatin accessibility, which may underlie the transcriptional dysregulation observed in mutant iPSCs. The greater number of DARs detected in MECP2^del6^ and MECP2^insA^ compared to MECP2^insT^ in our ATAC-seq results likely reflects the differential impact of each mutation on key MECP2 functional domains. MECP2 binds chromatin primarily through its MBD, NTD, and TRD [42]. The MBD is the core functional domain responsible for recognizing and binding symmetrically methylated CpG dinucleotides. This binding plays a crucial role in stabilizing chromatin structure, particularly in densely packed genomic regions such as heterochromatin [1, 43]. In addition to the MBD, the NTD possesses DNA-independent chromatin-binding capabilities and contributes significantly to chromatin structure and stability. It acts synergistically with the MBD to promote chromatin compaction and overall chromatin integrity [44]. The TRD, located at the C-terminus of MECP2, is essential for its function as a transcriptional repressor. It exerts its regulatory role by interacting with nucleosome remodeling complexes, thereby enhancing chromatin compaction and repressing transcriptional activity [45]. Together, these findings suggest that mutations affecting these domains, such as those in MECP2^del6^ and MECP2^insA^, are more likely to disrupt MECP2’s chromatin-binding and remodeling functions, resulting in broader changes in chromatin accessibility compared to MECP2^insT^.

Next, we annotated the DARs to determine their genomic context. For all three MECP2 mutations, more than half of the DARs were mapped to promoter regions and distal intergenic regions, the latter often corresponding to potential enhancer elements [46]. Specifically, in MECP2^del6^, 63.36% of DARs were annotated to promoters (38.88%) or distal intergenic regions (24.48%). In MECP2^insA^, this proportion was 54.53% (29.51% promoter + 25.02% intergenic), and in MECP2^insT^, 59.37% (32.90% promoter + 26.47% intergenic) (Fig. 5E and Supplementary Table 3). To investigate the common regulatory consequences of MECP2 mutations on chromatin accessibility, we extracted the DARs shared across all three mutant lines and annotated the corresponding 462 target genes. GO enrichment analysis was performed on these genes to explore their functional implications. In the GOBP category (Fig. 5F), the enriched pathways were consistent with our RNA-seq results, primarily involving development and differentiation, reinforcing the notion that MECP2 mutations modulate pluripotency in iPSCs by altering chromatin accessibility. In the GOMF category, we identified significant enrichment in histone H3K9me2 methyltransferase activity, GTPase regulator activity, and cadherin binding, indicating potential disruption of epigenetic regulation, signaling dynamics, and cell adhesion (Fig. 5G). Meanwhile, in the GOCC category, enrichment was observed in pathways related to the centrosome, perinuclear region of the cytoplasm, and cell–cell junctions (Fig. 5H), suggesting broader structural and signaling alterations in MECP2-mutant iPSCs. Interestingly, altered intercellular spacing has been frequently observed in neurons [47] compared to astrocytes [48] in patients with RTT. This phenomenon has been proposed as a model for classic synaptic deficits associated with MECP2 mutations [49]. Neurons harboring MECP2 mutations often exhibit reduced cell size and fewer synaptic connections, leading to a decrease in cell-to-cell contact frequency [50] and a corresponding reduction in the expression of neuronal adhesion molecules [51]. In the context of stem cells, cell–cell contact plays a critical role in regulating iPSC fate decisions, functioning through feedback loops that influence pluripotency and lineage commitment [52]. Furthermore, during differentiation, changes in mechanical properties of the cellular microenvironment mediated by cell contact can directly impact iPSC differentiation potential [53]. Taken together, our findings highlight how MECP2 mutations may disrupt iPSC fate and differentiation by modulating cell–cell interactions and adhesion-related signaling pathways, providing mechanistic insights from multiple complementary perspectives.

To investigate the regulatory implications of chromatin accessibility changes induced by MECP2 mutations, we conducted transcription factor binding motif analysis on the DARs. Remarkably, across all three MECP2 mutant lines, the transcription factor with the highest binding likelihood was OCT4, a core regulator of pluripotency (Fig. 5I). This finding aligns with our earlier observation that MECP2 mutations lead to an aberrant reduction of OCT4 expression during EB formation. In addition to OCT4, we also identified enrichment of SOX2 and SOX3 binding motifs within DARs. These results suggest that key pluripotency transcription factors remain functionally engaged in regulating gene expression even under MECP2-deficient conditions. However, their premature depletion in the context of MECP2 mutations may contribute to dysregulated downstream gene expression, disrupting the balance required for proper differentiation. Beyond pluripotency factors, we observed notable enrichment of CTCF and CTCFL motifs. CTCF typically binds to intronic and intergenic regions rather than promoter regions [54]. while CTCFL shows a preference for promoter regions, especially those containing CTCF-like motifs [55]. This strongly suggests that MECP2 mutations promote an open chromatin state that creates de novo binding opportunities for CTCF, a key architectural regulator of chromatin organization.

### MECP2 mutation in iPSC triggers enhanced binding of CTCF to chromatin

**Fig. 6:**
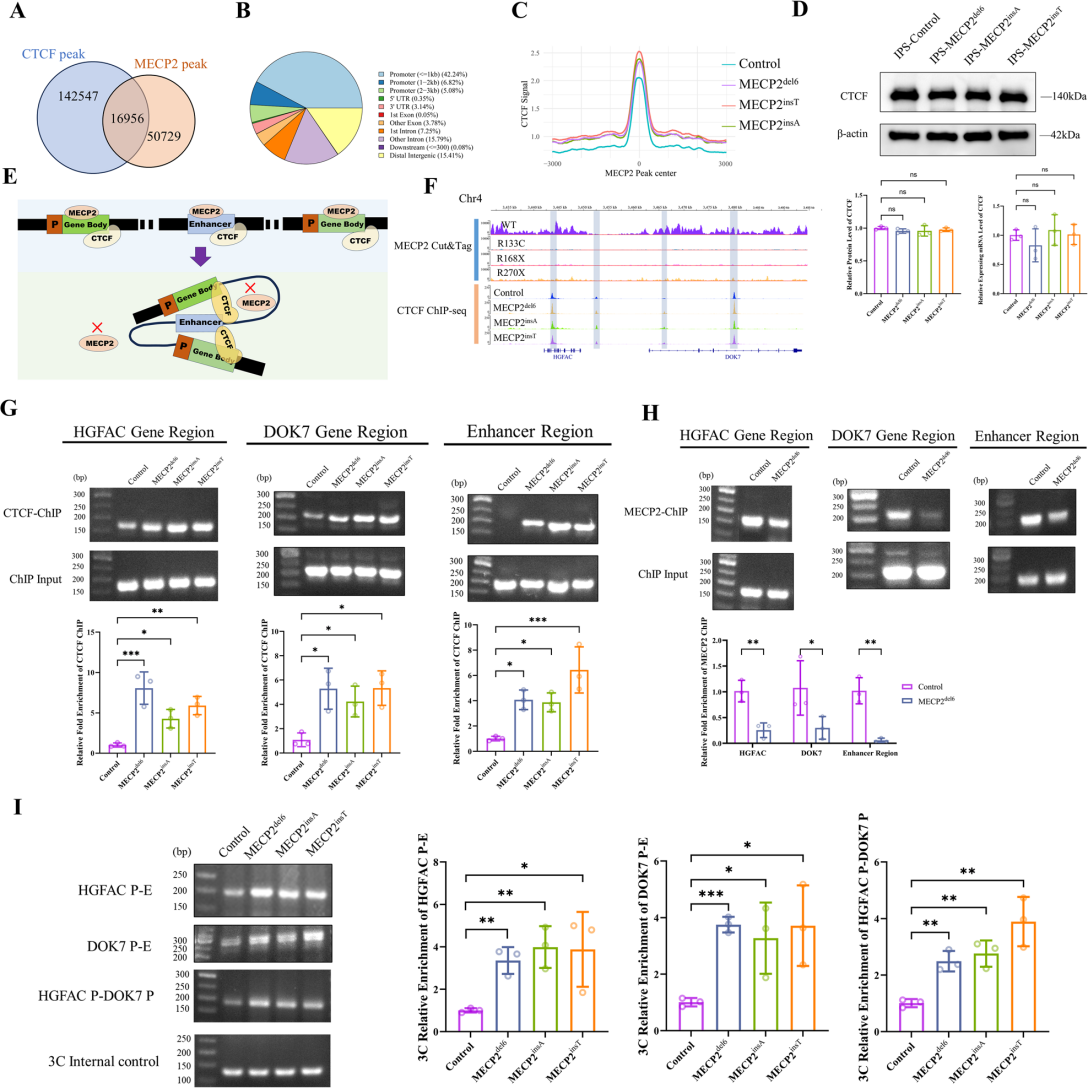
MECP2 mutations enhance CTCF binding and remodel 3D chromatin architecture. **A** Genome-wide co-localization analysis of CTCF and MeCP2 binding sites. We used the CTCF ChIP-Seq results from control IPS to compare with the CUT&TAG results from control ESC-derived neurons from Liu et al. **B** Genomic distribution of MECP2–CTCF co-bound sites. **C** Comparison of CTCF binding strength at MECP2–CTCF co-bound sites between control and MECP2 mutant iPSCs. **D** qPCR and western blot analysis of CTCF mRNA and protein levels in control and MECP2 mutant iPSCs. *n* = 3 biological replicates. **E** Schematic model illustrating the proposed relationship between MECP2 loss, enhanced CTCF occupancy, and altered chromatin organization. **F** Genome browser view of the HGFAC–DOK7 locus showing CTCF ChIP-seq signals and published MECP2 CUT&Tag profiles in control and MECP2 mutant conditions. **G** ChIP-qPCR validation of CTCF binding intensity at representative loci. Mutations in MECP2 enhance CTCF binding at these sites. *n* = 3 biological replicates. **H** ChIP-qPCR showing MECP2 binding changes at critical genomic sites in mutants. *n* = 3 biological replicates. **I** 3C confirms increased interaction frequencies at remodeled loci. *n* = 3 biological replicates. Data are presented as means ± SD, unpaired Student’s *t* test. *p < 0.05, **p < 0.01; ***p < 0.001; ****p < 0.0001.

The organization of the three-dimensional genome is a multilayered and dynamic process that integrates core loop extrusion mediated by CTCF and cohesin, scaffolding by tissue-specific proteins, compartmental anchoring to the nuclear lamina, and synergistic regulation by the transcriptional machinery and RNAs [56]. Collectively, these mechanisms ensure the spatiotemporal precision of gene expression, although different cell types and developmental stages may depend on distinct combinations of regulatory factors. A key unresolved question is whether CTCF, a central organizer of 3D genome architecture, also contributes to chromatin remodeling and accessibility in the context of MECP2 deficiency. Our ATAC-seq results suggested that MECP2 mutations might influence CTCF binding by altering chromatin accessibility. To directly test this hypothesis, we next performed CTCF ChIP-seq experiments. To address this, we performed CTCF ChIP-seq on control iPSCs and three MECP2 mutant iPSC lines (Supplementary Table 4). We attempted to perform MECP2 ChIP-seq in iPSCs using a ChIP-grade MECP2 antibody; however, consistent with previous reports [57–59], the resulting signal was extremely low and did not permit reliable peak calling. These technical limitations are in line with prior studies demonstrating that conventional ChIP-based approaches are insufficient for resolving MECP2 genome occupancy (Supplementary Table 5). Instead, we utilized high-resolution CUT&Tag data for MECP2 from neurons derived from hESCs [57]. Comparison of these MECP2 CUT&Tag profiles with our CTCF ChIP-seq data revealed colocalized binding sites of MECP2 and CTCF on chromatin (Fig. 6A). Notably, over half (54.14%) of these shared binding sites were located in promoter regions (Fig. 6B). Moreover, CTCF binding strength at these sites was enhanced in MECP2 mutant cells (Fig. 6C), suggesting a potential compensatory or regulatory interaction between CTCF and MECP2. We examined both mRNA and protein levels of CTCF in control and MECP2 mutant iPSCs and found no significant changes in CTCF expression following MECP2 mutation (Fig. 6D). Based on this, we hypothesize that reduced MECP2 binding, accompanied by increased CTCF occupancy at MECP2-CTCF co-bound sites in MECP2 mutants, may lead to CTCF-mediated alterations in chromatin spatial organization. These structural changes could, in turn, impact the expression of downstream genes through chromatin remodeling mechanisms (Fig. 6E). To test this hypothesis, we selected a representative genomic locus encompassing HGFAC (*chr4:3,441,005–3,449,486*) and DOK7 (*chr4:3,463,306–3,501,482*), two adjacent genes on chromosome 4 that share a common enhancer region. Integration of our CTCF ChIP-seq data with published MECP2-mutant Cut&Tag datasets revealed [57] that the increase in CTCF binding at these specific loci was accompanied by a loss of MECP2 binding (Fig. 6F). The promoters and the shared enhancer region of HGFAC and DOK7 coincide with co-binding sites of MECP2 and CTCF. We first conducted CTCF ChIP-qPCR at these regions and observed increased CTCF occupancy at the HGFAC promoter, DOK7 promoter, and the shared enhancer across all three MECP2 mutant iPSC lines (Fig. 6G), consistent with our bioinformatics findings. Due to the MECP2 ChIP-grade antibody recognizing the C-terminal region of MECP2, ChIP-qPCR against MECP2 was only feasible in Control and MECP2^del6^ lines. In these samples, MECP2 binding at the HGFAC promoter, DOK7 promoter, and the shared enhancer was reduced following MECP2 mutation (Fig. 6H), supporting the observed reciprocal relationship between MECP2 loss and CTCF gain at these regulatory regions. These findings confirmed that MECP2 mutations, which weaken MECP2 binding at sites co-occupied with CTCF, are associated with increased CTCF binding at the same loci. Given CTCF’s critical role in organizing 3D genome architecture, we next asked whether this enhanced binding could influence gene expression by altering chromatin spatial structure. To investigate this, we performed 3 C and observed increased interaction frequencies between the HGFAC promoter and its enhancer, the DOK7 promoter and its enhancer, as well as between the HGFAC and DOK7 promoters in MECP2 mutant cells (Fig. 6I). These results suggest that the upregulation of HGFAC and DOK7 expression (Fig. S6A) is likely driven by increased chromatin contacts, implicating CTCF-mediated chromatin remodeling as a downstream effect of MECP2 loss. To further determine whether MECP2 dysregulation directly leads to aberrant CTCF enrichment and CTCF-mediated chromatin structural rearrangements, we performed wild-type MECP2 complementation experiments in the MECP2^insA^ mutant iPSC background. Transfection with plasmids expressing exogenous wild-type MECP2 successfully restored MECP2 protein expression in MECP2^insA^ iPSCs (Fig. S7A). MECP2 complementation reduced the elevated transcription levels of HGFAC and DOK7 observed in MECP2^insA^ iPSCs (Fig. S7B). Because both genes exhibited enhanced CTCF binding in the mutant context, we further assessed CTCF occupancy at their promoter regions and shared enhancer elements using CTCF ChIP-qPCR. The results showed that the significantly increased CTCF binding signals in MECP2^insA^ iPSCs were attenuated following wild-type MECP2 restoration (Fig. S7C), indicating that abnormal CTCF enrichment at these regulatory sites is dependent on the absence of MECP2. In addition, we examined the spatial chromatin interaction patterns of the HGFAC–DOK7 locus using 3 C analysis. MECP2 complementation effectively reduced the abnormally enhanced promoter–enhancer and intergenic interactions observed in mutant cells (Fig. S7D), consistent with the normalization of CTCF binding signals.

We next analyzed genome-wide CTCF binding profiles in MECP2^del6^, MECP2^insA^, and MECP2^insT^ mutant iPSCs. Compared to control cells, all three mutants exhibited a marked global increase in CTCF binding, as evidenced by the widespread gain of CTCF peaks (Supplementary Table 4). Differential peaks were predominantly enriched in promoter and distal intergenic regions (Fig. S6B–D and Supplementary Table 4). Specifically, 219 differential peaks were identified in MECP2^del6^, with 185 showing increased binding; MECP2^insA^ exhibited 1954 differential peaks, of which 1843 were enhanced; and MECP2^insT^ displayed 582 differential peaks, including 538 with increased CTCF occupancy. This finding corresponds to the global contact enhancement observed in Hi-C. We further compared TAD change sites with CTCF peaks and found that 12.85% of TAD alterations overlapped with CTCF differential peaks (Fig. S6E). Previous studies have demonstrated that TADs can be formed independently of CTCF [60]. For example, in mammals, a subset of TAD boundaries overlaps with actively transcribed genes but not with CTCF binding sites, suggesting that CTCF is not universally required for TAD formation [61]. Our results indicate that MECP2 mutations also influence the subset of TADs formed through CTCF regulation. Since FIRE formation depends in part on the adherens in complex and CTCF [34]. We next compared specific FIREs with CTCF differential peaks. We found that more than half of the MECP2^insA^-specific FIREs overlapped with CTCF differential peaks (32/56), whereas Control-specific FIREs rarely overlapped with them (1/565) (Fig. S6F). These findings suggest that MECP2 mutations induce changes in chromatin spatial organization that are, at least in part, mediated by CTCF.

In conclusion, MECP2 mutations enhance CTCF binding at MECP2-CTCF co-occupied sites, leading to CTCF-mediated reorganization of chromatin architecture and subsequent changes in downstream gene expression.

## Discussion

Although MECP2 has been extensively studied, there is a lack of multi-omics analyses in pluripotent stem cells, and its role as an organizer of higher-order chromatin structure within the three-dimensional genome of pluripotent stem cells remains largely unknown. In this study, we provide evidence that MECP2 influences cell fate decisions in iPSCs through chromatin remodeling and spatial genome reorganization. Our findings suggest that the effects of MECP2 on 3D genome structure may be mediated, at least in part, by its interactions with other architectural proteins, such as CTCF, which are known to regulate chromosomal topology.

Using CRISPR-Cas9 genome editing, we generated three distinct MECP2 mutant lines: MECP2^del6^, MECP2^insA^, and MECP2^insT^ from male iPSCs, each targeting one of the three structural domains of MECP2: the MBD, TRD, and CTD (Fig. 1A–D). We observed that MECP2 mutations negatively impacted iPSC proliferation and growth (Figs. 1G and S1E). While previous studies have suggested that MECP2 may not be essential for stem cell maintenance [62]. No prior research has specifically addressed how MECP2 mutations affect the growth dynamics of iPSCs. To assess differentiation potential, we conducted EB formation assays. Although MECP2 mutations did not impair the capacity of iPSCs to differentiate into the three germ layers (Fig. S2A–D), they did alter the morphology and structural integrity of EBs, particularly affecting their growth rate (Fig. 2). EBs are self-organizing aggregates of pluripotent stem cells that recapitulate key aspects of early embryogenesis. As such, they serve as valuable in vitro models for investigating the molecular mechanisms underlying early developmental processes [63]. While previous studies have shown that MECP2 mutations do not impair the ability of iPSCs to differentiate into the three germ layers [64], their impact on EB formation and structure remains unclear. Clinically, MECP2 mutations have been associated with placental abnormalities, impaired stem cell differentiation, and defective embryonic development, which may ultimately lead to miscarriage [65, 66]. In mouse models, chimeric embryos containing MECP2-deficient cells exhibit severe developmental abnormalities, with the severity correlating with the proportion of mutant cells [67]. Similarly, in primate models such as rhesus monkeys, MECP2 mutations have been shown to cause late-term miscarriage, reflecting embryonic developmental failure reminiscent of human Rett syndrome pathology [68]. Our findings provide supporting evidence that MECP2 plays a crucial role in stem cell proliferation and early embryonic development, as reflected by altered EB morphology and growth in MECP2-mutant iPSCs.

The precise folding of the three-dimensional genome, including the organization of chromatin compartments and TADs, is essential for establishing and maintaining cellular identity [69]. Our Hi-C analyses show that MECP2 loss of function induces profound, multilayered remodeling of 3D genome architecture in human pluripotent stem cells. At the global level, we observed changes in the distance-decay pattern of chromatin interaction frequencies (Fig. S3A), and at the compartment level, more than 40% of genomic regions exhibited A/B compartment switching (Fig. S3C, D). This extensive reprogramming highlights MECP2 as a critical factor in maintaining the global epigenetic state of pluripotent stem cells, extending its previously described role in promoting compartmental segregation in neurons [70] to the undifferentiated stage, and revealing an overall shift of genomic activity towards a more active state. At the subregional compartmental scale, MECP2 mutations induced extensive remodeling of TAD boundaries, including boundary gains, losses, and shifts (Fig. 3A–F). Although these structural changes were not universally linked to differential expression of neighboring genes at the global level (Fig. S4E), their functional consequences were strongly dependent on cell-type-specific contexts [71, 72]. Notably, genes near TADs with boundary loss were significantly down-regulated and enriched in neurodevelopmental pathways (Fig. 3I), directly linking MECP2 mutations to their core neurological phenotypes. By contrast, genes near boundary-gaining TADs were preferentially up-regulated and enriched in fundamental processes such as the cell cycle (Fig. 3H), consistent with the proliferation abnormalities observed in MECP2-deficient cells [73]. This suggests that TAD remodeling induced by MECP2 mutation may ‘predetermine’ regulatory errors at the pluripotent stem cell stage, whose full impact becomes progressively unmasked during subsequent differentiation.

Analysis of chromatin local interaction hotspots (FIREs/super-FIREs) revealed that MECP2 mutation leads to significant reprogramming of specific FIREs, possibly by perturbing enhancer–promoter interactions that regulate key gene expression [34]. Notably, super-FIRE analysis identified 199 regions specifically acquired in MECP2 mutants, within which genes were significantly enriched for those associated with chromatin remodeling complexes such as SWI/SNF (Fig. 4J). The SWI/SNF complex, an ATP-dependent chromatin remodeler, establishes chromatin accessibility in regulatory regions by repositioning nucleosomes [74, 75], and its malfunction is strongly linked to developmental disorders and cancers [76, 77]. These findings suggest that MECP2 may influence local chromatin accessibility by regulating super-FIRE formation. In contrast, control-specific super-FIREs were primarily associated with basic cellular homeostasis and adhesion (Fig. 4K), highlighting the role of MECP2 in safeguarding the three-dimensional genome under physiological conditions.

Dynamic regulation of chromatin accessibility is a key epigenetic mechanism that determines cell identity and fate. Our ATAC-seq analyses confirmed that MECP2 loss-of-function triggers extensive and mutation severity–dependent chromatin accessibility remodeling in iPSCs (Fig. 5A–D). Among the mutants, MECP2^insT^ induced relatively limited changes, consistent with the established role of MECP2 in synergistically binding and compacting chromatin through its MBD, NTD, and TRD domains [78]. Regions showing altered accessibility were significantly enriched in promoters and putative enhancers (Fig. 5E), with associated genes clustered in pathways related to development and differentiation (Fig. 5G, H). Motif analysis further revealed a prominent enrichment of binding sites for core pluripotency factors such as OCT4 and SOX2/SOX3 in these differentially accessible regions (Fig. 5I). The pluripotency network is a highly interconnected and self-sustaining feedback system, in which OCT4, SOX2, and NANOG not only regulate downstream targets but also reinforce their own expression via autoregulation and mutual activation [79]. This observation provides a mechanistic explanation for the premature downregulation of OCT4 during EB differentiation in MECP2 mutants and suggests that the pluripotency network may aberrantly remodel the chromatin landscape through feedback regulation. Finally, enrichment of the CTCF/CTCFL motif further connects altered chromatin accessibility to the activity of this major three-dimensional genome organizer.

Alterations in the binding kinetics of CTCF, a key organizer of the three-dimensional genome [80], are directly involved in the destabilization of TAD boundaries caused by MECP2 mutations and the reprogramming of local interaction hotspots. In this study, we found that MECP2 mutation significantly enhanced CTCF binding across the genome, particularly at promoter regions (Fig. 6A–C), without changes in CTCF expression levels (Fig. 6D). At the HGFAC/DOK7 locus, we observed that reduced MECP2 binding coincided with enhanced CTCF occupancy, increased chromatin looping strength, and upregulation of gene expression (Fig. 6F–I). These results reveal a potential antagonistic relationship between MECP2 and CTCF in human pluripotent stem cells, in contrast to the synergistic effect reported in differentiated cells [81], highlighting the cell-type and locus-specific nature of MECP2 function. The distinctive dynamic chromatin architecture of pluripotent stem cells, together with possible competition between MECP2 and CTCF at hemi-methylated CpG sites [82], likely contributes to the complexity of this interaction. Importantly, these findings converge on a unifying model in which MECP2 loss in pluripotent cells disrupts higher-order chromatin organization through coordinated effects on both local interaction FIREs/super-FIREs and architectural protein occupancy. By reshaping super-FIRE landscapes and relieving constraints on CTCF binding at key regulatory regions, MECP2 deficiency may prime aberrant chromatin looping and transcriptional programs at an early developmental stage. Such early architectural dysregulation provides a plausible mechanistic framework linking MECP2 mutations to the initiation of RTT pathogenesis long before overt neuronal differentiation and clinical symptom onset.

Together, these findings establish a novel mechanistic framework in which MECP2 functions as a global regulator of the three-dimensional genome in pluripotent stem cells. MECP2 safeguards chromatin accessibility and ensures the proper functioning of the pluripotency gene network by maintaining high-level chromatin architecture, fine-tuning local interaction hotspots, and dynamically balancing the activity of genome organizers such as CTCF. Mutations in MECP2 induce multilevel disruptions, affecting both global spatial architecture and local regulatory elements, particularly altering the topology of key neurodevelopmental genes. These changes ultimately lead to aberrant cell fate decisions through misregulated chromatin accessibility. This work provides a new three-dimensional genomic perspective for understanding Rett syndrome pathogenesis and offers a theoretical foundation for future therapeutic strategies aimed at restoring chromatin structure.

## Method and materials

### Ethics approval

The project was approved by Xi’an Jiaotong University Ethics in Health Research (No.2022-1149). All methods were performed in accordance with the relevant guidelines and regulations. The human iPSC lines used in this study were derived from donor samples obtained with written informed consent as described in our previous study [20].

### iPSCs maintenance

All iPSC lines, including both gene-edited and control lines, were maintained under feeder-free conditions following established protocols [83]. Briefly, iPSCs were cultured on Matrigel-coated plates (Corning, 354277) in Essential 8 medium (Cellapy, #CA1014500). Cells were passaged every 5–7 days using 0.5 mM EDTA (Thermo Fisher, 15575-038). Routine monitoring for mycoplasma contamination and karyotypic abnormalities was performed to ensure the genomic integrity and health of the cell lines. The iPSC lines used in this study were generated and fully characterized in our previous study [20].

### CRISPR Cas9 gene edit

For gene editing, the pSpCas9(BB)-2A-Puro (PX459) V2.0 plasmid (Addgene plasmid #62988) was used following established protocols [84]. Guide RNAs were designed using the CRISPOR online tool (http://crispor.tefor.net/), with target sequences selected based on the human genome assembly GRCh38/hg38 (December 2013). Cas9-gRNA plasmids were transfected into iPSCs using Lipofectamine 3000 (Thermo Fisher Scientific), and cells were collected 48 h post-transfection for genotyping. iPSCs were transfected with the target vector and cultured for 48 h. Following transfection, cells were cultured in E8 medium supplemented with 0.3 μg/mL puromycin for 48 h to select for puromycin-resistant cells. After puromycin selection, cells were cultured in puromycin-free E8 medium for 5 days. Cells were plated on Matrigel-coated 6-well plates at a density of 500 cells per well in E8 medium supplemented with 10 μM Y-27632 ROCK inhibitor (MCE, HY-10071). After 48 h, the inhibitor was removed, and cells were maintained in E8 medium for 10 days before being transferred to 24-well plates for clonal expansion. Gene-edited iPSC clones were identified and confirmed by Sanger sequencing as previously described [20]. Potential off-target sites were predicted using Cas-OFFinder [85] and validated through Sanger sequencing.

### Cell proliferation assays

Control and MECP2-mutant iPSCs were dissociated using Accutase (Thermo Fisher, A11105-01) and seeded at a density of 2000 cells per well in Matrigel-coated 96-well plates. After allowing cells to adhere for 4 h, CCK-8 reagent (Yeasen, 40203ES60) was added to each well and incubated for an additional 4 h. Optical density (OD) at 450 nm was measured using a microplate reader to assess cell viability. Measurements were taken at the same time each day over a 5-day period to monitor cell proliferation dynamics.

### Colony formation assay

Control iPSCs and MECP2-mutant iPSCs were dissociated using Accutase (Thermo Fisher, A11105-01), and 2000 cells were seeded into each well of a Matrigel-coated 6-well plate. Cells were cultured in E8 medium supplemented with 10 μM Y27632 for the first 2 days. Thereafter, the medium was replaced with E8 medium without Y27632 and refreshed every 3 days for an additional 10 days. At the end of the culture period, colonies were fixed with 4% paraformaldehyde for 30 min at room temperature and stained with 0.2% crystal violet for 30 min. Three biological replicates were included per group.

### Embryoid body formation

iPSCs exhibiting >80% confluency were gently dissociated using 0.5 mM EDTA (Thermo Fisher, 15575-038) for 10 min at room temperature. After carefully aspirating the dissociation solution, 3 mL of Essential 6 medium (Cellapy, CA1017500) was added to each well. Cell clumps were dislodged using a sterile silicone spatula and transferred into a 6-well plate pre-rinsed with Anti-Adherence Rinsing Solution (STEMCELL Technologies, 07010) using a 5 mL pipette. To quantify EB growth, iPSCs were dissociated using Accutase and incubated for 10 min at 37 °C. Cells were then centrifuged at 100 × *g* for 3 min and resuspended in E6 medium supplemented with 10 μM Y27632. A total of 10,000 cells per well were seeded into ultra-low attachment U-bottom 96-well plates (Thermo Fisher, 174925). Plates were centrifuged at 100 × *g* for 3 min to aggregate the cells into the well centers and incubated at 37 °C in a 5% CO₂ atmosphere. After 24 h, EBs were gently transferred into 6-well plates pre-rinsed with Anti-Adherence Rinsing Solution (STEMCELL Technologies, 07010) for further culture and growth monitoring. The culture medium was replaced every other day to support embryoid body formation.

### Frozen section

EBs were collected on day 7 of differentiation, washed twice with PBS, and fixed in 4% paraformaldehyde (PFA) overnight at 4 °C. Following fixation, samples were incubated in 30% sucrose solution for 4 h for cryoprotection, then embedded in OCT compound (Leica, 14020108926). Frozen sections were prepared using a frozen sectioning machine (Leica CM1950) at a thickness of 10–15 μm.

### Immunofluorescence

Cells were fixed in 4% PFA for 15 min at room temperature, followed by permeabilization with 0.2% Triton X-100 in PBS for 10 min. After washing, samples were blocked in 3% bovine serum albumin (BSA) for 1 h at room temperature. Primary antibodies, diluted in 3% BSA, were applied and incubated overnight at 4 °C. The next day, samples were washed with PBS and incubated with appropriate fluorescently labeled secondary antibodies for 1 h at room temperature in the dark. Frozen sections were stained using the AlphaTSA™ Multiplex IHC Kit for Tissue (Alpha X Biotech, AXT37100031) according to the manufacturer’s instructions. Briefly, slides were first rinsed with PBS to remove residual OCT compound, then incubated with the primary antibody diluent for 15 min at room temperature to block non-specific binding. Sections were incubated with the primary antibody, diluted in the same diluent, for 1 h at 37 °C. After washing, HRP-conjugated secondary antibody was applied and incubated for 10 min at 37 °C. Signal amplification was performed using fluorescent dye-containing tyramide solution for 5 min at room temperature. Antigen retrieval between successive antibody staining rounds was conducted using AbEraser (Alpha X Biotech, AXT9700500) to remove bound antibodies and enable subsequent staining. The following primary antibodies were used: SOX2 (Rabbit, 1:500, Abcam, ab92494), OCT4 (Rabbit, 1:500, Abcam, ab19857), TRA-1-60 (Mouse, 1:500, Abcam, ab16288), SSEA4 (Mouse, 1:500 Abcam, ab16287), AFP (Mouse, 1:200, ABclonal, A17898), α-SMA (Rabbit, 1:200, ABclonal, A1011), TUBB3 (Rabbit, 1:200, ABclonal, A17913), ZO-1 (Rabbit, 1:100, Abmart, TA5145). The following secondary antibodies were used: Alexa Fluor® (1:1000, Abcam, ab150077, ab150113, ab150080, ab150116). All samples were mounted with ProLong™ Glass Antifade Mountant (Thermo Fisher, P36982).

### Protein extraction and western blotting

Total proteins were extracted from tissues and cells with RIPA lysis buffer (Beyotime, P0013B) containing a protease inhibitor cocktail (MCE, HY-K0010). Protein concentration was determined using a BCA Protein Assay Kit (Beyotime, P0010). Equal amounts of protein lysates were separated on 10% SDS‑PAGE gels and transferred onto methanol‑activated PVDF membranes (Roche, 03010040001). The membranes were blocked with 5% non‑fat milk in TBST for 1 h at room temperature and then incubated overnight at 4 °C with primary antibodies. After washing, the membranes were incubated with appropriate horseradish peroxidase (HRP)-conjugated secondary antibodies for 1 h at room temperature. Protein bands were detected using an ECL chemiluminescence kit (PCM, PC-30002) and visualized with a ChemiDoc™ Touch imaging system (Bio‑Rad, USA). The following primary antibodies were used: MECP2 (C‑terminal) (Rabbit, 1:2000, Abcam, ab195393), MECP2 (N‑terminal) (Rabbit, 1:1000, Abcam, ab2828), CTCF (Rabbit, 1:1000, Abcam, ab128873), and beta Actin (Mouse, 1:20,000, Proteintech, 66009‑1‑Ig). HRP‑conjugated secondary antibodies included Goat Anti‑Rabbit IgG (1:10,000, Abways, AB0101) and Goat Anti‑Mouse IgG (1:10,000, Abways, AB0102).

### RNA extraction and quantitative real-time PCR

Total RNA was extracted from cell lines and frozen tissue samples using TRIzol reagent (Genestar, P118-05) following the manufacturer’s instructions. RNA concentration and purity were determined using a DeNovix spectrophotometer (USA). First-strand cDNA was synthesized using the Hifair® II 1st Strand cDNA Synthesis SuperMix for qPCR (gDNA digester plus) (Yeasen, 11123ES60). Quantitative real-time PCR was performed using the Hieff® qPCR SYBR Green Master Mix (Yeasen, 11201ES08) according to the manufacturer’s protocol. Reactions were conducted in triplicate on an iQ5 Multicolor Real-Time PCR Detection System (Bio-Rad, USA). ACTB served as the internal control. The primers used were as follows: ACTB-F, 5′-CACCATTGGCAATGAGCGGTTC-3′; ACTB-R, 5′-AGGTCTTTGCGGATGTCCACGT-3′; OCT4-F, 5′-CGAAAGAGAAAGCGAACCAG-3′; OCT4-R, 5′-GCCGGTTACAGAACCACACT；CTCF-F, 5′-GACCACACAAGTGCCATCTCTG-3′; CTCF-R, 5′- ATGTCGCAGTCTGGGCACTTGT-3′; DOK7-F, 5′-GCCATCATGCTGGGCTTTGACA-3′; DOK7-R, 5′- AACTTGGTGCCTGGAGCCACTG-3′; HGFAC-F, 5′-TGGAACTCCGATCTGCTCTACC-3′; HGFAC-R, 5′- CCTCTCGTCATTGTCCGGATTC-3′.

### Chromosome conformation capture (3C)

Cells were harvested, resuspended in medium, and filtered through a 40 μm strainer. Crosslinking was performed using 1% formaldehyde by adding 27 μL of 37% stock solution per 10 mL sample at room temperature for 15 min. The reaction was quenched with 600 μL of 2.5 M glycine and incubated for 30 min at room temperature before centrifugation at 225 × *g* for 8 min at 4 °C. The cell pellet was lysed in 5 mL of ice-cold lysis buffer containing 10 mM Tris-HCl, pH 7.5, 10 mM NaCl, 5 mM MgCl₂, 0.1 mM EGTA, and 1× complete protease inhibitor for 10 min, followed by centrifugation at 1000 × *g* for 5 min at 4 °C. Chromatin was digested in 500 μL of 1.2× buffer with 7.5 μL of 20% SDS at 37 °C for 1 h, then treated with 50 μL of 20% Triton X-100 at 37 °C for 1 h. Restriction enzyme digestion was performed using 200–300 units of enzyme PstI(Neb, R3140S) at 37 °C overnight. The reaction was stopped by adding 40 μL of 20% SDS to achieve a final concentration of 1.6% and incubating at 65 °C for 30 min. For ligation, the reaction mixture was adjusted to 6.45 mL with 750 μL of 10× buffer and 5.7 mL water, followed by the addition of 375 μL of 20% Triton X-100 and incubation at 37 °C for 1 h. Ligation was performed with 8 μL of ligase (NEB, B020S) at 16 °C for 8 h. After adding 300 μg of Proteinase K (Beyotime, ST532) and incubating at 65 °C overnight, RNA was removed with 300 ng of RNase at 37 °C for 1 h. DNA was extracted with 5 mL of phenol-chloroform, precipitated with sodium acetate and ethanol, washed with 70% ethanol, and dissolved in 50–100 μL of TE buffer for qRT-PCR analysis. RT-PCR products were subjected to electrophoresis on a 2% agarose gel to verify amplicon size and specificity. The primers used were as follows: HGFAC-Promoter, 5′-GGAGGGGAGCAGAAGAGGA-3′; DOK7-Promoter, 5′- ATGTCGCAGTCTGGGCACTTGT-3′, Enhancer, 5′-ACAGCTGCCAGTATCCCCTA-3′; ABCA13-P, 5′- GGAGTAATCGGAGCAGCTACC -3′; ABCA13-E, 5′- AGAAACTGAGCAAGGCCTGA -3′.

### RNA-seq

Cells were washed twice with 1× PBS and collected in 1 mL of Trizol following the manufacturer’s instructions. RNA quality was assessed using an Agilent TapeStation system, and only samples with an RNA Integrity Number greater than 8.4 were selected for library preparation. Poly(A)-enriched libraries were prepared by Annoroad Gene Technology (Beijing) and sequenced on the Illumina NovaSeq platform using 150 bp paired-end reads, with a sequencing depth of approximately 30 million reads per replicate.

RNA-seq data were processed using the ENCODE standardized pipeline for long RNAs. Ribosomal RNA sequences were first removed from raw reads using SortMeRNA. The remaining reads were aligned to the human reference genome GRCh38 using STAR with two-pass mode for improved splice junction detection. Gene-level quantification was performed using RSEM with GENCODE v35 annotations, generating transcripts per million (TPM) and expected read counts. Quality control metrics included mapping rate, rRNA contamination level, and library complexity assessed via duplication rates. Strand specificity was verified using RSeQC. For differential expression analysis, raw counts were normalized using DESeq2. All analyses adhered to ENCODE quality standards, including the use of biological replicates and verification of replicate concordance through Pearson correlation. Detailed protocols are available on the ENCODE portal (https://www.encodeproject.org/data-standards/rna-seq/long-rnas/).

### ATAC-seq

ATAC-seq libraries were prepared following standard protocols. Briefly, cells were collected and lysed to isolate nuclei. The nuclei were then incubated with Tn5 transposase to simultaneously fragment and tag accessible chromatin regions. After DNA purification, the fragments were amplified by PCR with indexed primers to construct sequencing libraries. Library quality was assessed using Qubit (Thermo Fisher) and Bioanalyzer (Agilent), and qualified libraries were pooled based on concentration. Sequencing was performed on the Illumina NovaSeq 6000 platform (PE150) at Annoroad Gene Technology (Beijing).

ATAC-seq data were processed using the ENCODE ATAC-seq pipeline. Briefly, paired-end reads were aligned to the reference genome (GRCh38) using Bowtie2, and mitochondrial reads were removed. Duplicates were marked with Picard, and peaks were called using MACS2. Tn5 transposition bias was corrected, and blacklisted regions were filtered. Quality control metrics included the fraction of reads in peaks and Tn5 enrichment. BigWig signal tracks were normalized for visualization. Detailed protocols are available on the ENCODE portal (https://www.encodeproject.org/atac-seq/). Motif enrichment analysis was performed using HOMER [86]. and DEGs were annotated with genomic features using ChIPseeker [87].

### Chromatin immunoprecipitation (ChIP)

Cells were cross-linked with 1% formaldehyde for 15 min at room temperature and quenched with 125 mM glycine. Chromatin was fragmented by sonication to an average size of approximately 200 bp. The lysates were incubated overnight at 4 °C with specific primary antibodies, followed by capture of DNA-protein complexes using Dynabeads Protein A/G (Invitrogen, 88803). Reverse cross-linking was carried out by incubation with sodium chloride at 65 °C for 7 h. DNA was subsequently purified using phenol-chloroform/isoamyl alcohol extraction. ChIP-enriched DNA was analyzed by quantitative real-time PCR and agarose gel electrophoresis. The primers used were as follows: HGFAC-chip-F, 5′- GGCTCAGGGGTCACTCAAC -3′; HGFAC-chip-R, 5′- CTCACTTCCAGGCTGCAGAA -3′; DOK7-chip-F, 5′- CACCTTTCTCCTCCTTGCGT -3′; DOK7-chip-R, 5′- GGAGACATTGAGGAACGTGGT -3′; Enhancer Region-F, 5′- GGGTGATGCAGGTTCAGGAA -3′; Enhancer Region-R, 5′- GAGTGTGCGTGTGAATGCTC -3′;

### ChIP-seq

DNA fragments were obtained through ChIP as described. Library fragment size distribution was analyzed using the Agilent 5400 system to verify DNA integrity and confirm the expected insert size profile. Sequencing was performed on Illumina PE150 to generate high-quality data for downstream analysis. ChIP-seq high-throughput sequencing was performed by Novogene (Beijing, China).

ChIP-seq data were processed using the ENCODE Transcription Factor ChIP-seq pipeline. Briefly, raw reads were aligned to the reference genome (GRCh38) using Bowtie2. Duplicate reads were removed using Picard, and peaks were called with MACS2 (FDR < 0.05). Blacklisted regions were filtered out. For quality control, metrics such as PCR bottlenecking coefficient and cross-correlation analysis were assessed. Replicate concordance was evaluated using the Irreproducible Discovery Rate framework. Signal tracks were generated as fold-change over control. Detailed protocols are available on the ENCODE portal (https://www.encodeproject.org/chip-seq/transcription-factor/). Annotate differential peaks using ChipSeeker [87].

### HI-C

Fresh cells were counted and resuspended in 10 mL complete medium, then fixed with 2% formaldehyde 571 μL of 37% solution for 10 min at room temperature with gentle mixing. The fixation was quenched by adding 894 μL of 2.5 M glycine for 10 min at RT, followed by 15 min on ice. Cells were pelleted 400 × *g* for 5 min at 4 °C, washed twice with PBS, and stored at −80 °C in sealed tubes. Each sample contained ≥1.0 × 10^6^ cells. Libraries were constructed by Novogene(Beijing) using qualified samples with Agilent 5400 Illumina sequencing. Hi-C data processing was performed in accordance with the ENCODE Hi-C Data Standards and Processing Pipeline to ensure data quality and consistency(https://www.encodeproject.org/hic/).

To visualize the hic matrix, we used Juicebox [88] to visualize large-scale chromatin contact heatmaps (>100 kb). For higher-resolution at 25 kb, 10 kb, and 5 kb, contact matrices were extracted with the straw package [88] in R and subsequently visualized using R.

### Topologically associating domains (TADs)

Differential TAD boundaries between control and MECP2^insA^ Hi-C contact matrices were identified using TADCompare [89]. SpectralTAD was first employed to define TAD boundaries, which were then compared across genotypes. Regions with an absolute differential boundary score greater than 2 and a *p* value < 0.05 were classified as differential boundaries. Gene annotation near these differential boundaries was performed using the GREAT tool with the following association parameters: basal + extension [25] (5 kb upstream, 1 kb downstream, and up to 100 kb maximum extension).

### Frequent interaction regions (FIREs)

Frequently interacting regions (FIREs) were called using the FIREcaller [39]. In brief, we used FIRECaller’s built-in method to directly process .hic files via Juicer Tools, operating at a 10 kb resolution. Differential FIREs were identified as follows: genomic regions with Control FIRE scores greater than qnorm (0.975) and MECP2^insA^ FIRE scores lower than qnorm(0.9) were defined as Control-specific FIREs. Conversely, MECP2^insA^-specific FIREs were defined as genomic regions with MECP2^insA^ FIRE scores greater than qnorm (0.975) and Control FIRE scores lower than qnorm (0.9) [25]. Control- and MECP2^insA^-specific super-FIREs were determined by filtering out super-FIREs shared between the two samples at the genome coordinate level. We also defined common FIREs as those detected in both Control and MECP2^insA^ iPSCs, with a FIRE score difference between the two cell types of less than 0.5 [35]. To link differential FIREs and super-FIREs to genes, we used the annotatr package [90]. To intersect FIRE and super-FIRE regions with all genic annotations, including 1–5 kb upstream of the TSS, promoter (<1 kb upstream of the TSS), 5′UTR, first exons, exons, introns, CDS, and 3′UTR.

### Gene cluster analysis and visualization

We used the Metascape [91] website (https://metascape.org/gp/index.html) for Gene Ontology (GO) enrichment analysis and Kyoto Encyclopedia of Genes and Genomes (KEGG) enrichment analysis. WEB-based GEne SeT Analysis Toolkit website [92] (https:// www.webgestalt.org/) and GSEA4.3.3 software [93] (UC SanDiego & BROAD Institute) for gene set enrichment analysis (GSEA). WashU Epigenome Browser (https://epigenomegateway.wustl.edu/) was used for HI-C and ChiP-seq visualization. We used SRplot [94] (https://www.bioinformatics.com.cn) for statistical computing.

### Statistics and reproducibility

Data analysis and visualization were performed using GraphPad Prism version 9.5.0 (GraphPad Software, Inc.). Normality of data distribution was assessed using the built-in normality tests in GraphPad Prism. Unless otherwise stated, data are presented as mean ± standard deviation. Statistical significance was determined using Student’s *t* test. A *p* value less than 0.05 was considered statistically significant. Significance levels are indicated as follows: *p < 0.05, **p < 0.01, ***p < 0.001, and ****p < 0.0001. Results that do not reach statistical significance are denoted as ns (not significant). Statistical details of the experiments can be found in the corresponding figure legends.

## Supplementary information

supplement

Checklist

original gel pictures

Supplement Table 1

Supplement Table 2

Supplement Table 3

Supplement Table 4

Supplement Table 5

## Data Availability

The data supporting the findings of this study can be obtained from the corresponding author upon reasonable request.
