# Supplementary material for: MECP2 mutations disrupt pluripotent stem cell fate through remodeling of the three-dimensional genome
Source: Cell Death Dis. 2026 May 8;17(1):609. doi: 10.1038/s41419-026-08837-4 (PMC13324534; doi:10.1038/s41419-026-08837-4)
Supplement: Supplementary file 1 — supplement [file 41419_2026_8837_MOESM1_ESM.docx]

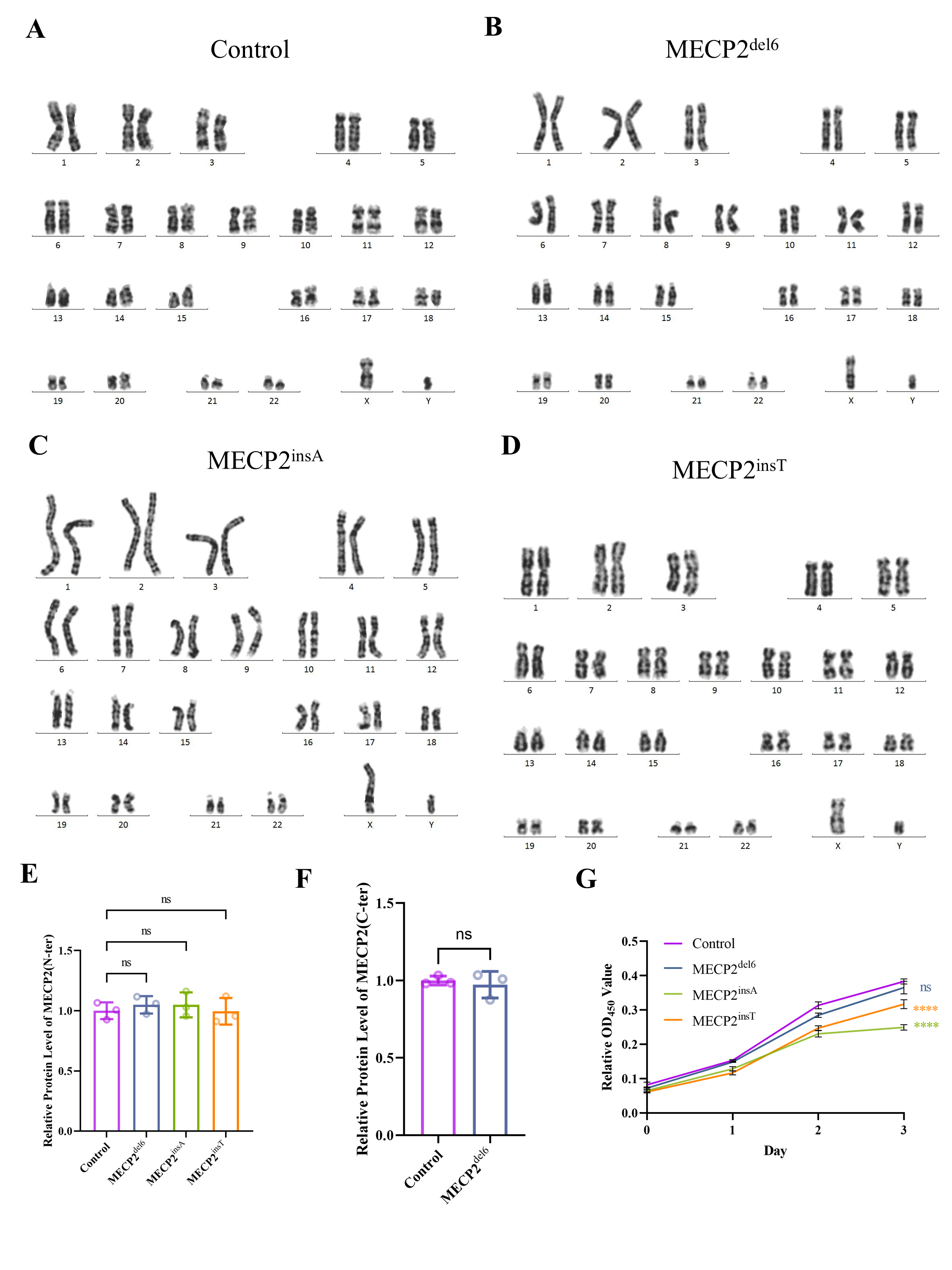


**Figure S1 Characterization of control and MECP2 mutant iPSCs**

A−D. Karyotype analysis confirmed a normal 46, XY chromosomal constitution in control iPSCs, MECP2^del6^, MECP2^insA^, and MECP2^insT^ mutant lines.

E. Western blot quantification results for MECP2 N-terminal specific antibodies. β-actin was used as the reference. *n* = 3 biological replicates.

F. Western blot quantification results for MECP2 C-terminal specific antibodies. β-actin was used as the reference. *n* = 3 biological replicates.

G. Proliferation assays showed significantly reduced growth rates in all MECP2 mutant iPSC lines compared to controls. *n* = 3 biological replicates. Data are presented as means ± SD, unpaired Student’s t test. ns = not significant; **P* < 0.05, ***P* < 0.01; ****P* < 0.001; *****P* < 0.0001


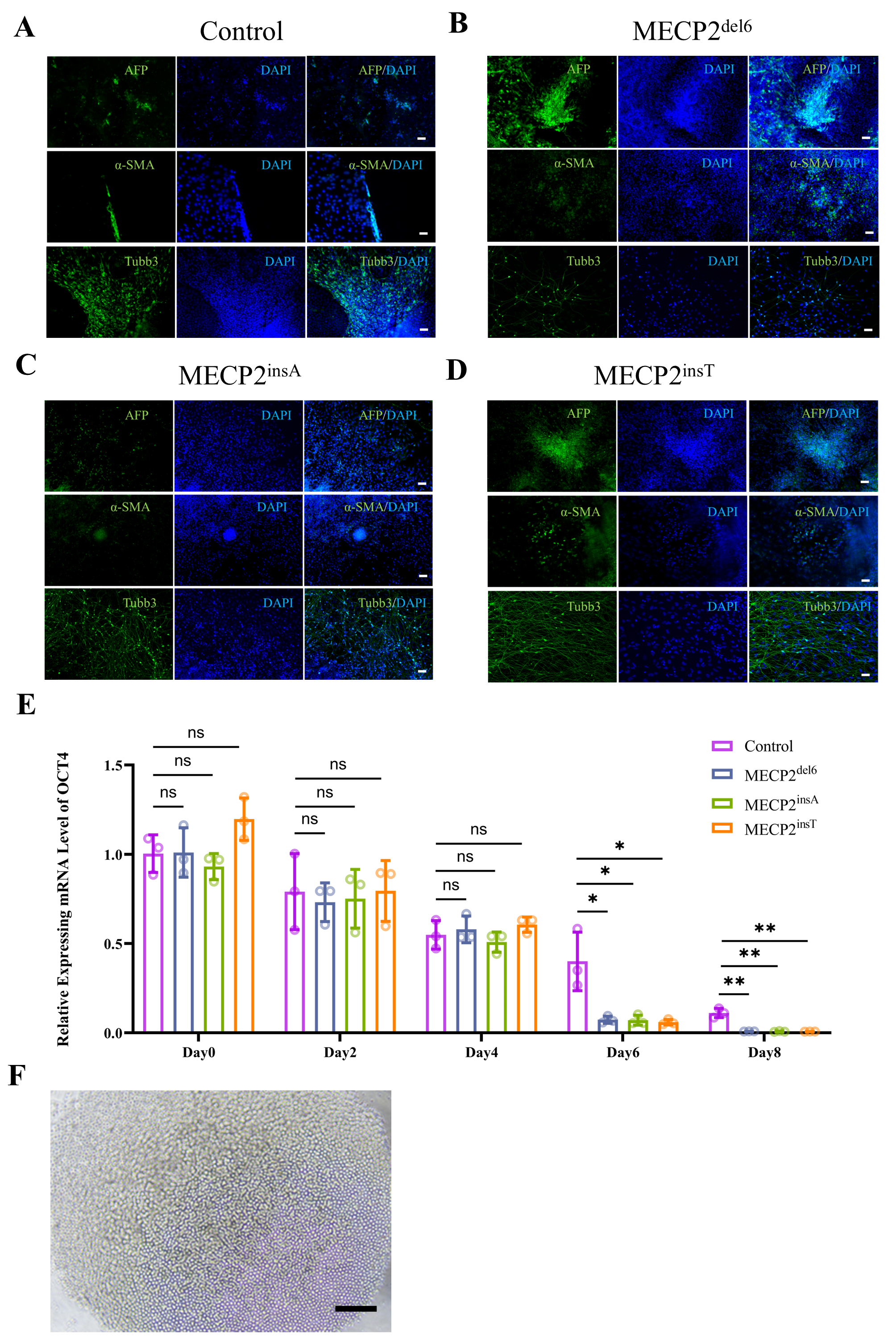


**Figure. S2 Characterization of control and MECP2 mutant iPSCs during early differentiation**

A−D. EBs were generated by differentiating iPSCs in Essential 6 medium for 7 days, followed by adherent culture for 3 days. Immunofluorescence staining was used to detect lineage-specific markers: ectoderm (TUBB3), mesoderm (α-SMA), and endoderm (AFP). Both control and MECP2 mutant iPSCs successfully differentiated into cell types representing all three germ layers. Scale bar = 50 μm.

E. qPCR analysis of OCT4 expression in EBs (day 0–8) showed a significant decrease in all MECP2 mutants relative to control from day 6 onward. *n* = 3 biological replicates. Data are presented as means ± SD, unpaired Student’s t test. ns = not significant; **P* < 0.05, ***P* < 0.01

F. iPSCs were dissociated into single cells and seeded into U-bottom 96-well plates, followed by centrifugation at 1000g for 3 minutes to promote uniform aggregation and minimize inter-sample variability.


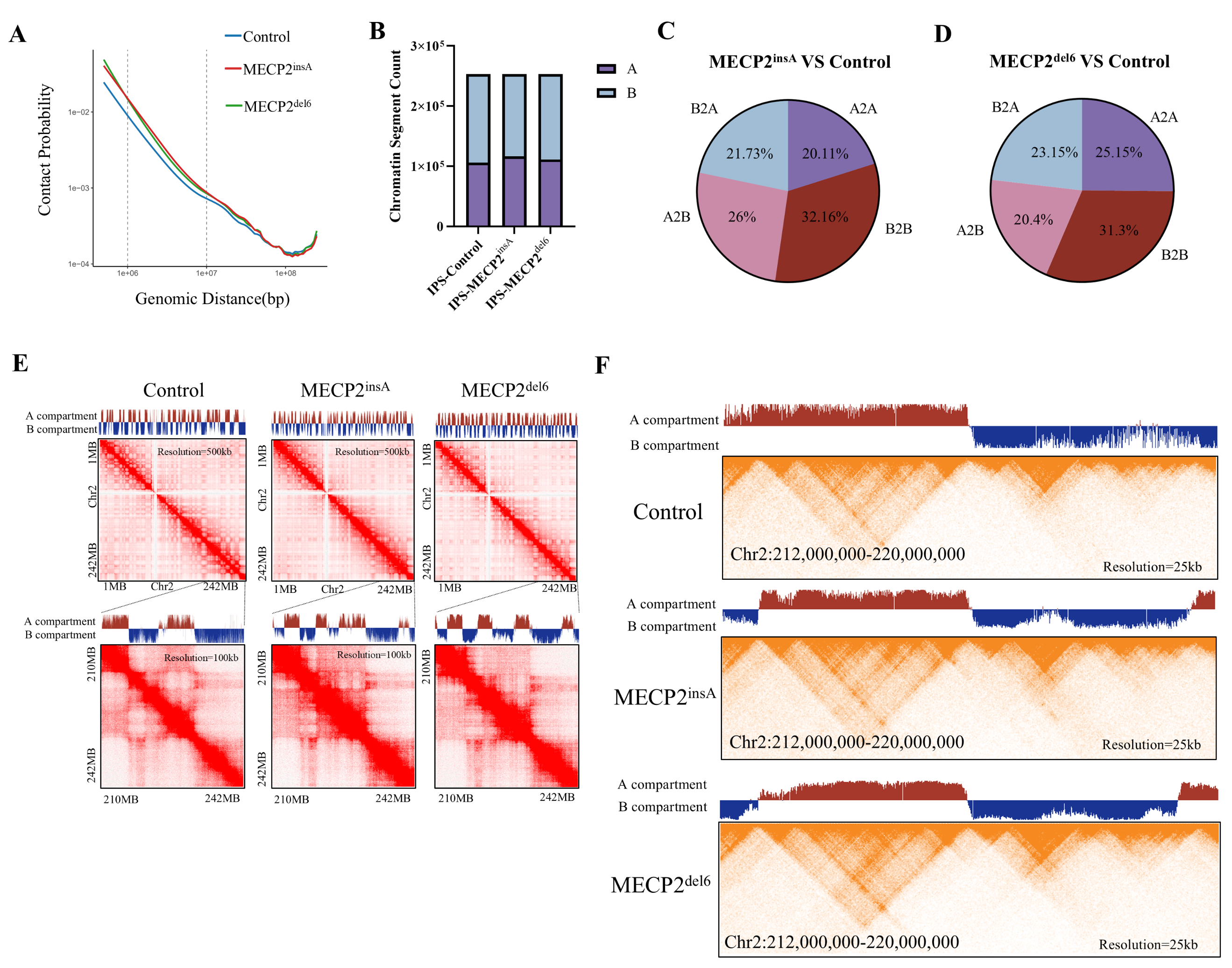


**Figure S3 Hi-C analysis reveals 3D genome reorganization in MECP2 mutants**

A. Distance-dependent decay of chromatin contact probability in control, MECP2^insA^, and MECP2^del6^ iPSCs derived from Hi-C data.

B. Genome-wide proportions of A and B compartments in control and MECP2 mutant iPSCs.

C–D. Compartment switching analysis comparing control with MECP2^insA^ (C) and MECP2^del6^ (D), showing transitions between A and B compartments.

E. Representative Hi-C contact maps at 500 kb (top) and 100 kb (bottom) resolution for control, MECP2^insA^, and MECP2^del6^ iPSCs, with corresponding A/B compartment tracks.

F. Representative Hi-C contact maps at 25 kb resolution for a selected genomic region (*chr2:212,000,000–220,000,000*) in control, MECP2^insA^, and MECP2^del6^ iPSCs, with corresponding A/B compartment profiles shown above each map.


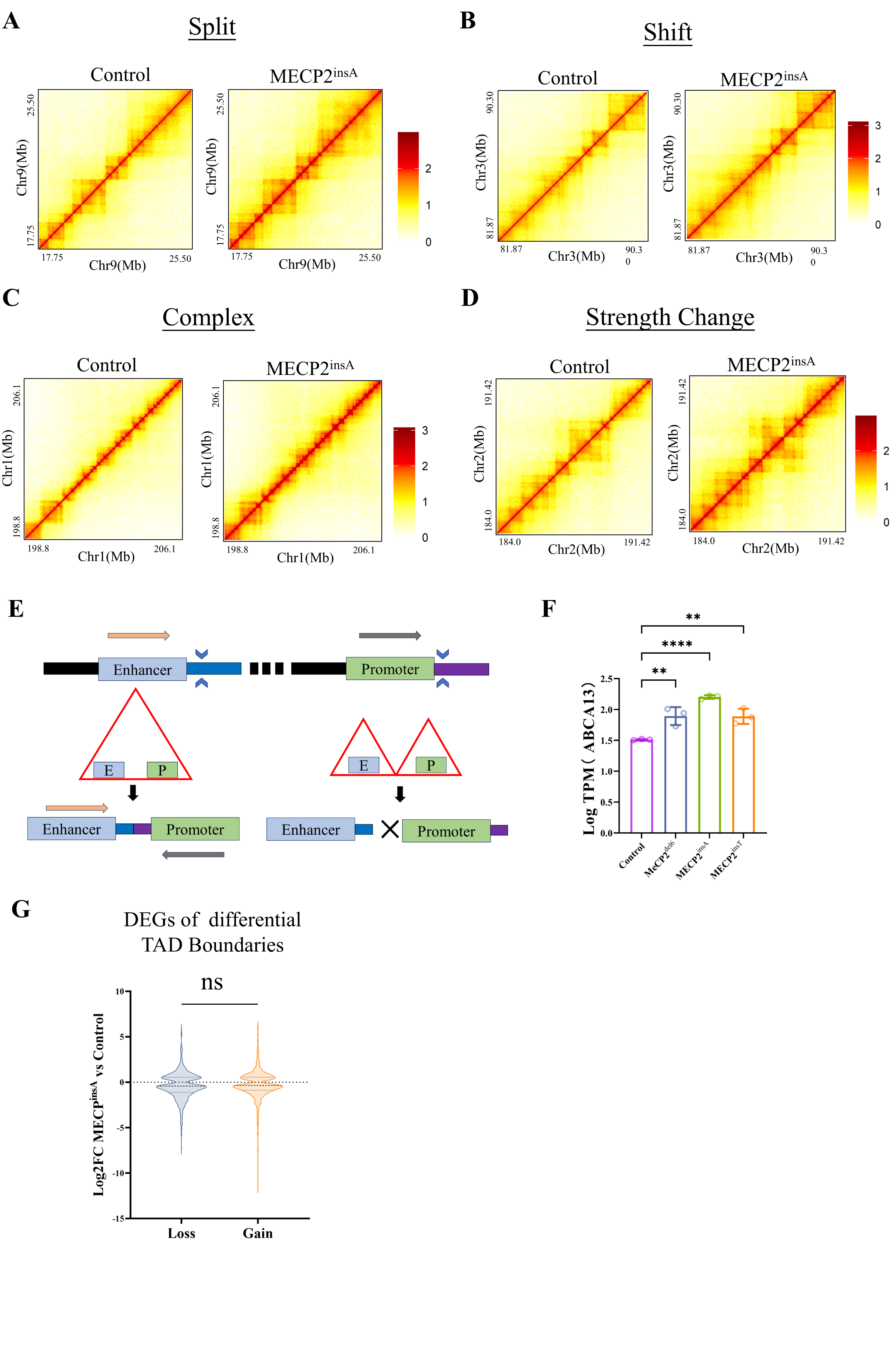


**Figure S4 MECP2 regulates the formation of TAD boundaries**

A. Contact heat map of representative regions in TAD Split, *chr9:17,750,000*–*25,500,000.*

B. Contact heat map of representative regions in TAD Shift, *chr3:81,875,000*–*90,300,000.*

C. Contact heat map of representative regions in TAD Complex, *chr1:198,800,000*–*206,100,000.*

D. Contact heat map of representative regions in TAD Strength Change, *chr2:184,000,000*–*191,425,000.*

E. Schematic diagram illustrating the principle of the 3C assay.

F. Expression of *ABCA13* in RNA-seq data. *n* = 3 biological replicates. Data are presented as means ± SD, unpaired Student’s t test. ns = not significant; ***P* < 0.01, ****P < 0.0001

G. Gene expression near differential boundaries. No significant differences in gene expression were observed for genes located near gained or lost TAD boundaries.


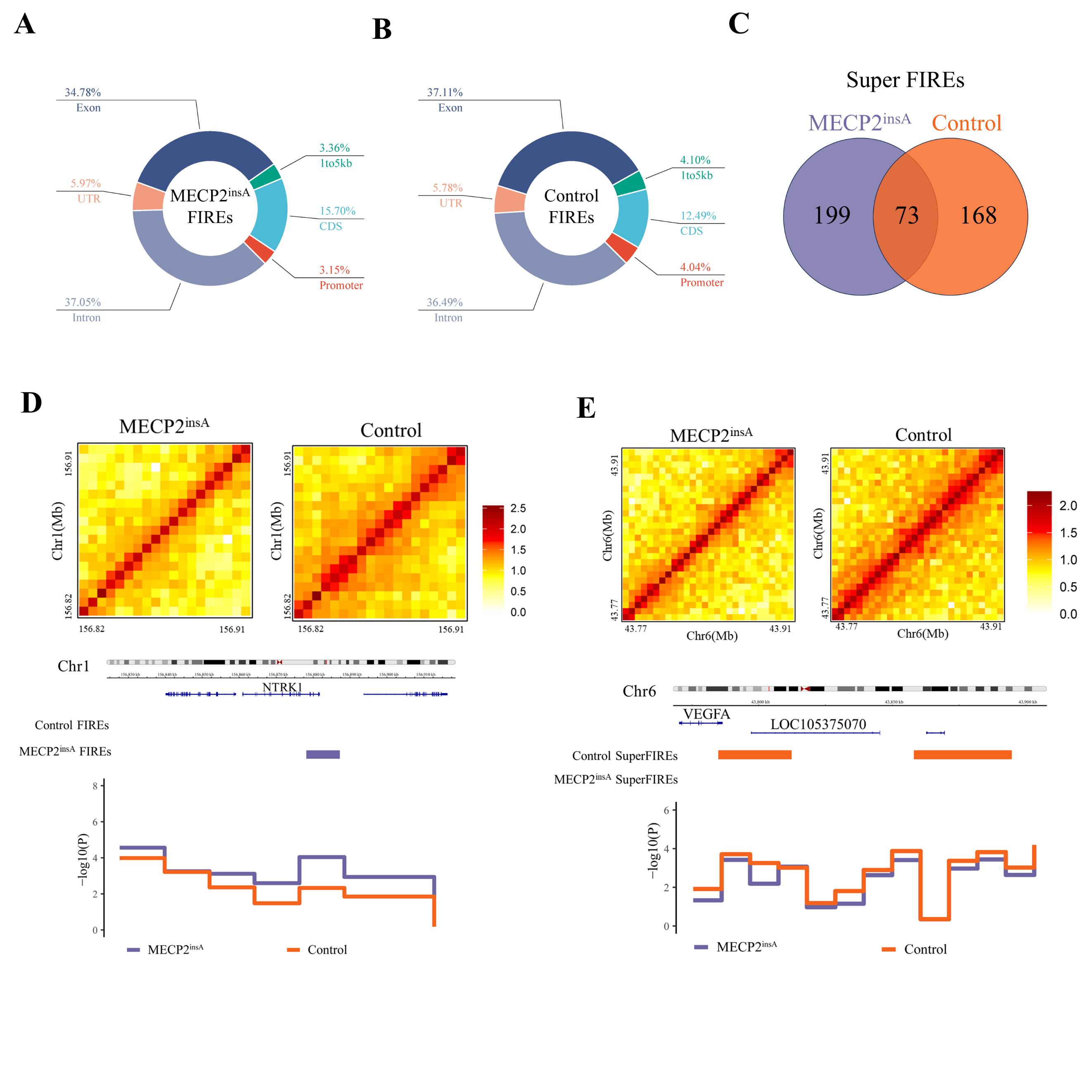


**Figure S5 Characterization of genotype-specific super-FIREs.**
A–B. Genomic annotation of the genomic regions underlying MECP2^insA^-specific FIREs (A) and Control-specific FIREs (B). The majority are located in gene bodies (Exons + Introns) and intergenic regions.

C. Venn diagram showing the number of common and genotype-specific super-FIREs identified in Control and MECP2^insA^ iPSCs.

D. Representative regions of MECP2^insA^-specific FIREs *chr1:156,824,140*–*156,918,576.*

E. Representative regions of control-specific super-FIREs *chr6:43,767,899*–*43,907,828.*


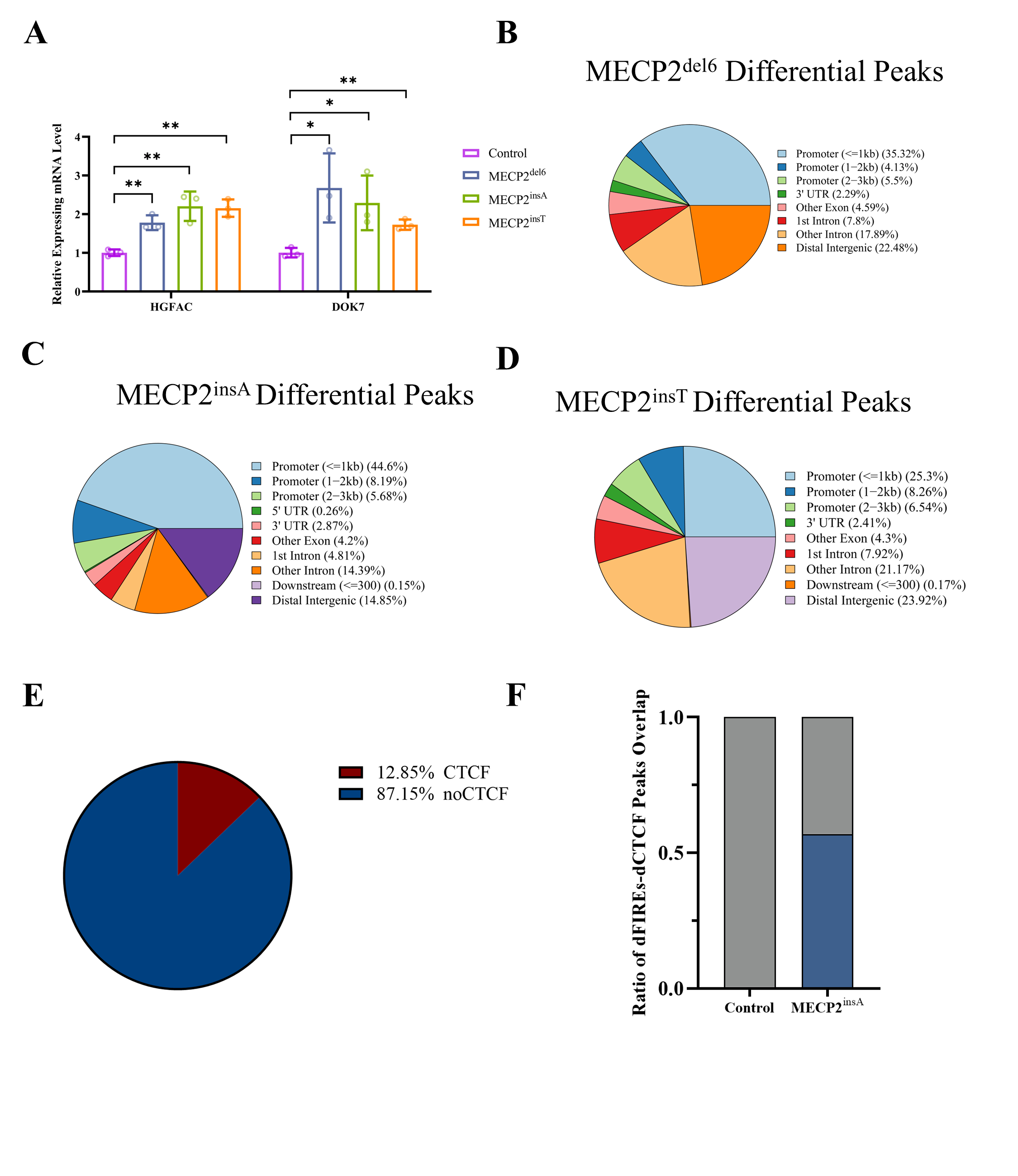


**Figure S6 CTCF binding profile analysis in MECP2 mutant iPSCs**

A. qPCR validation of *HGFAC* and *DOK7* expression levels in MECP2 mutant iPSCs compared to control. Expression was normalized to housekeeping genes and presented relative to control. *n* = 3 biological replicates. Data are presented as means ± SD, unpaired Student’s t test. ns = not significant; **P* < 0.05, ***P* < 0.01

B–D. Genomic distribution of differential CTCF binding peaks in MECP2^del6^, MECP2^insA^, and MECP2^insT^ mutant iPSCs. Peaks were annotated to genomic features such as promoters, introns, exons, and intergenic regions.

E. CTCF co-localization analysis. A total of 12.85% of differential TAD boundaries overlapped with CTCF binding peaks.

F. The bar chart shows the overlap between genotype-specific FIREs and differentially regulated CTCF binding peaks. Gray indicates MECP2-independent, blue indicates MECP2-dependent.


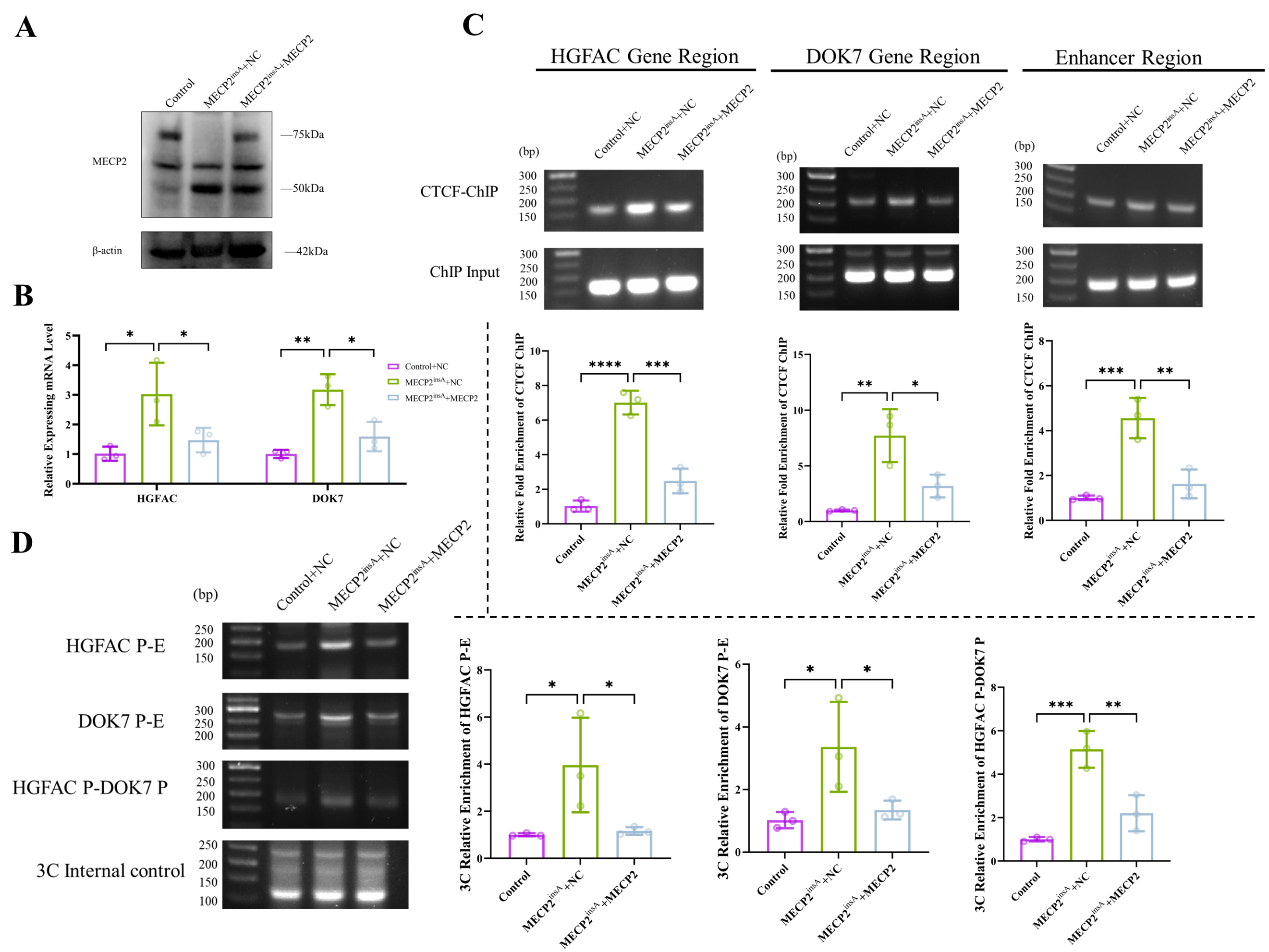


**Figure S7 Wild-type MECP2 complementation reverses aberrant CTCF binding and chromatin interactions in MECP2^insA^ iPSCs**

A. Western blot analysis showing restoration of MECP2 protein expression in MECP2^insA^ iPSCs following wild-type MECP2 complementation.

B. qPCR analysis of HGFAC and DOK7 expression in control, MECP2^insA^, and MECP2^insA^ cells complemented with wild-type MECP2. *n* = 3 biological replicates.

C. CTCF ChIP-qPCR analysis at the HGFAC promoter, DOK7 promoter, and their shared enhancer region in the indicated iPSC lines. Representative gel images (top) and quantification of CTCF enrichment relative to input (bottom) are shown. *n* = 3 biological replicates.

D. Chromatin conformation capture (3C) analysis of promoter–enhancer and intergenic interactions at the HGFAC–DOK7 locus. Representative PCR products (left) and relative interaction frequencies quantified by qPCR (right) are shown. *n* = 3 biological replicates. Data are presented as means ± SD, unpaired Student’s t test. ns = not significant; **P* < 0.05, ***P* < 0.01; ****P* < 0.001; *****P* < 0.0001
