# Supplementary material for: MECP2 mutations disrupt pluripotent stem cell fate through remodeling of the three-dimensional genome
Source: Cell Death Dis. 2026 May 8;17(1):609. doi: 10.1038/s41419-026-08837-4 (PMC13324534; doi:10.1038/s41419-026-08837-4)

Figure1F Original western blot image

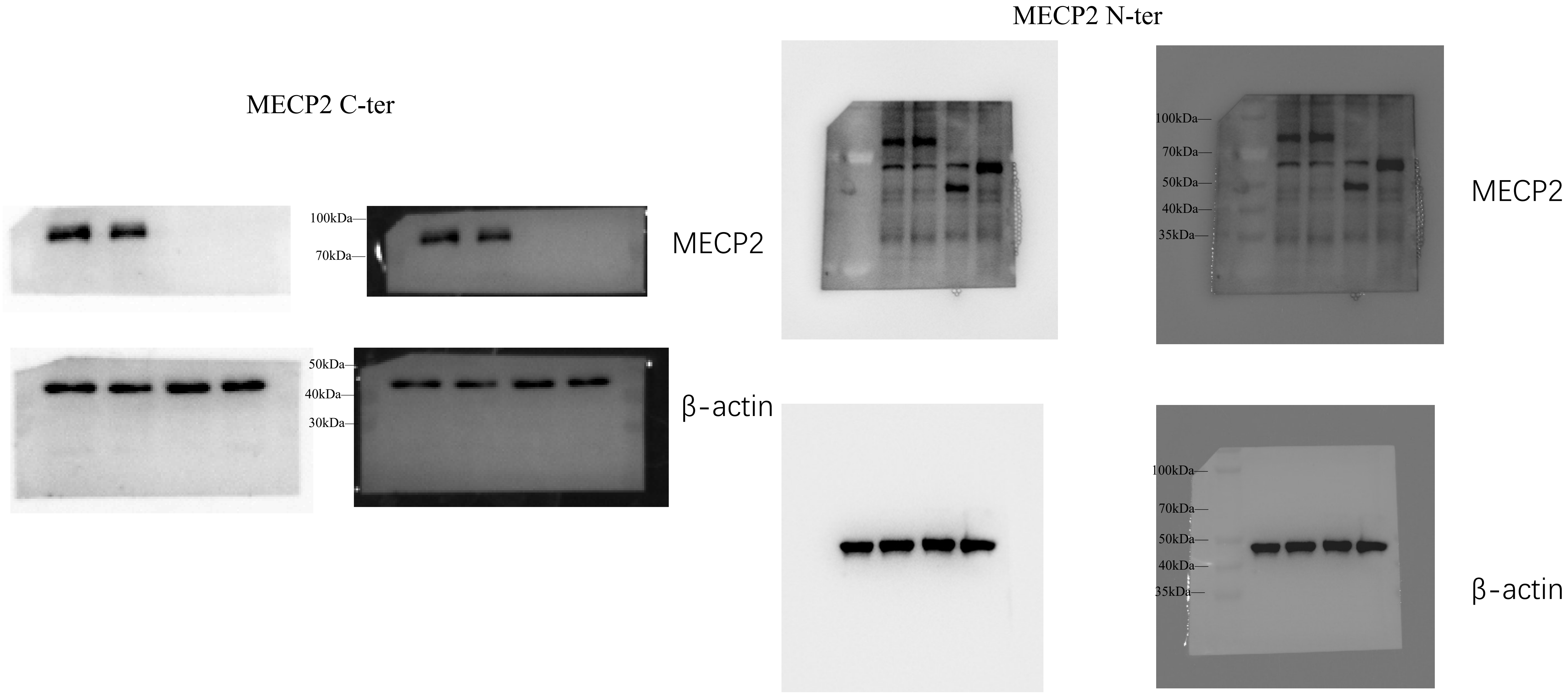

Figure 3H Raw agarose gel electrophoresis image of 3C products

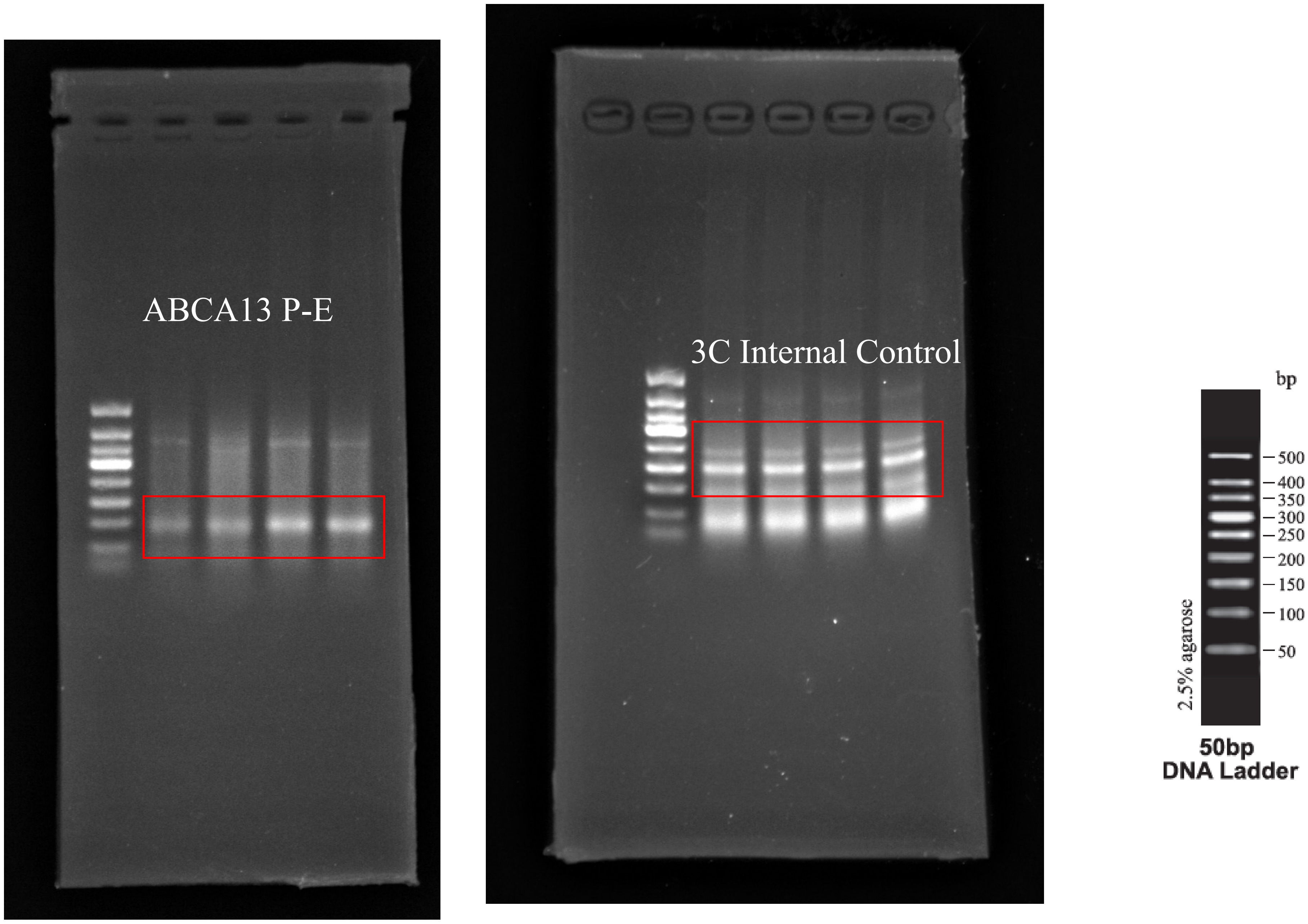

Figure6D Original western blot image

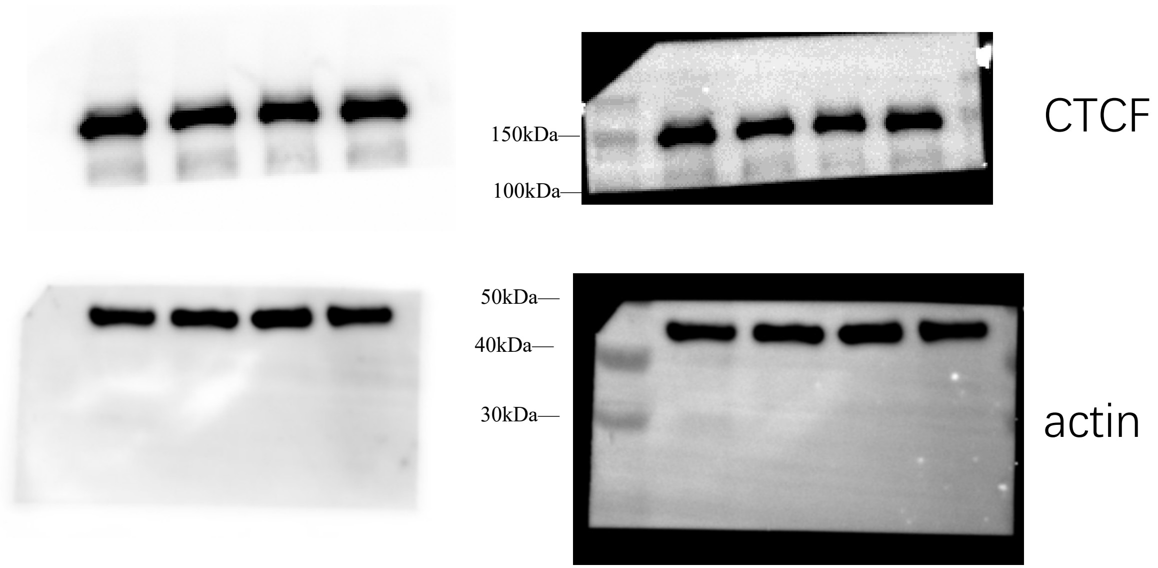

Figure 6G Raw agarose gel electrophoresis image of ChIP-qPCR products

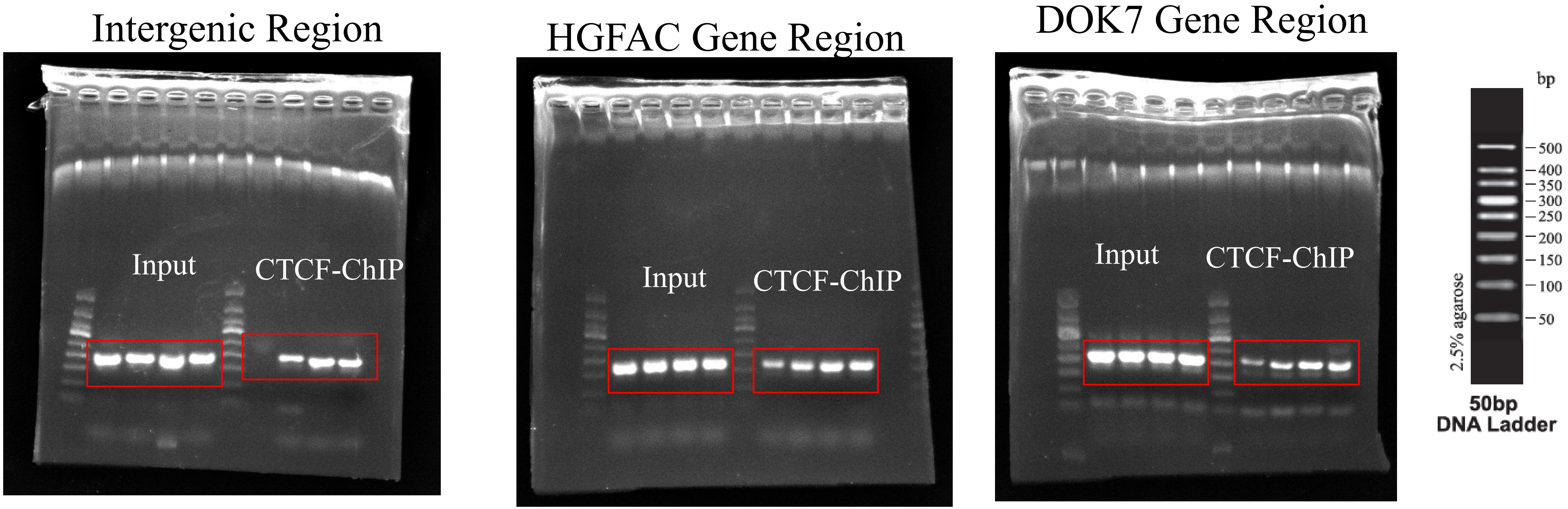

Figure 6H Raw agarose gel electrophoresis image of ChIP-qPCR products

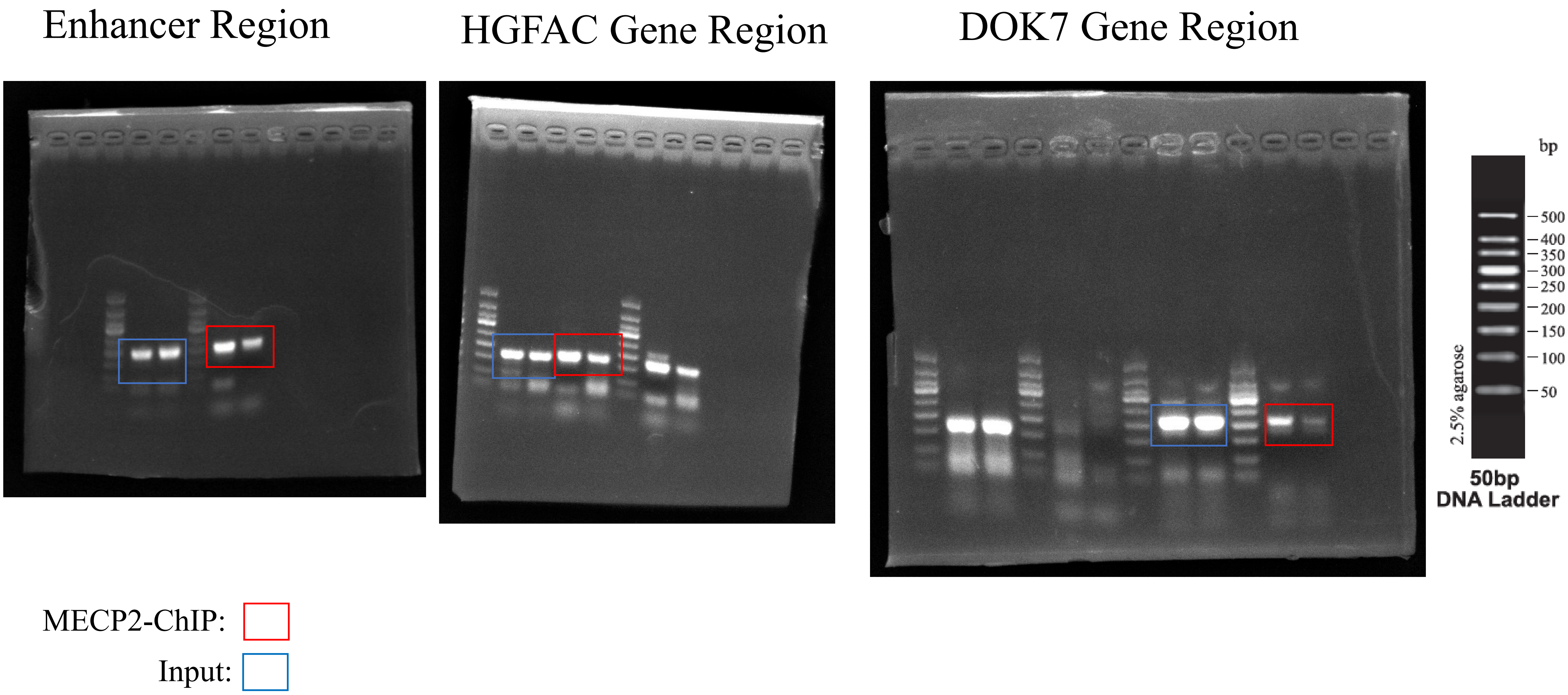

Figure 6I Raw agarose gel electrophoresis image of 3C products

HGFAC P-E

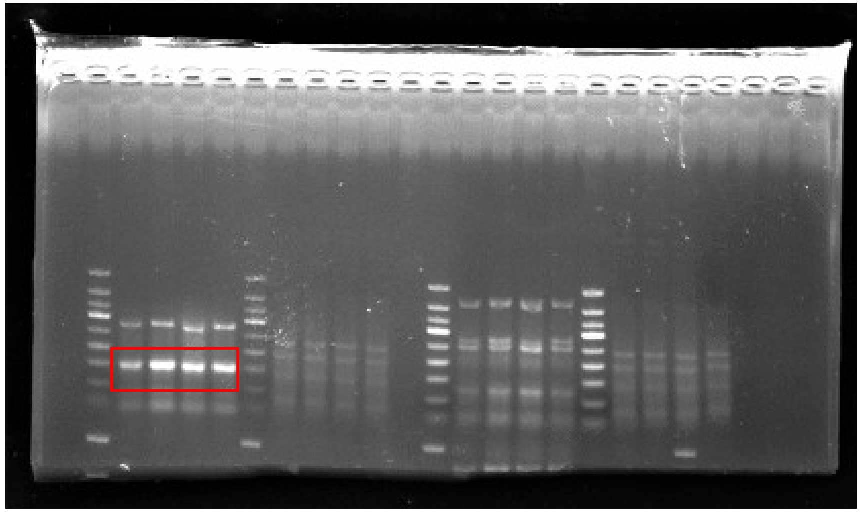

DOK7 P-E

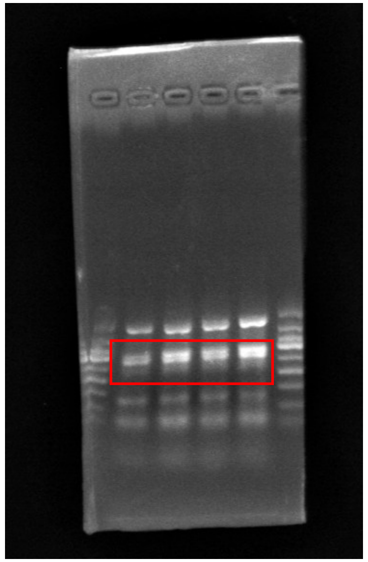

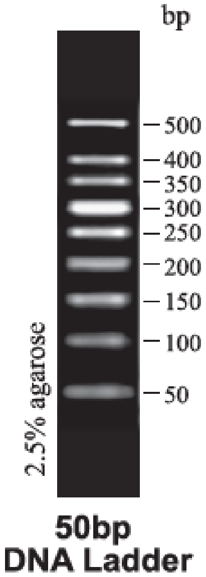

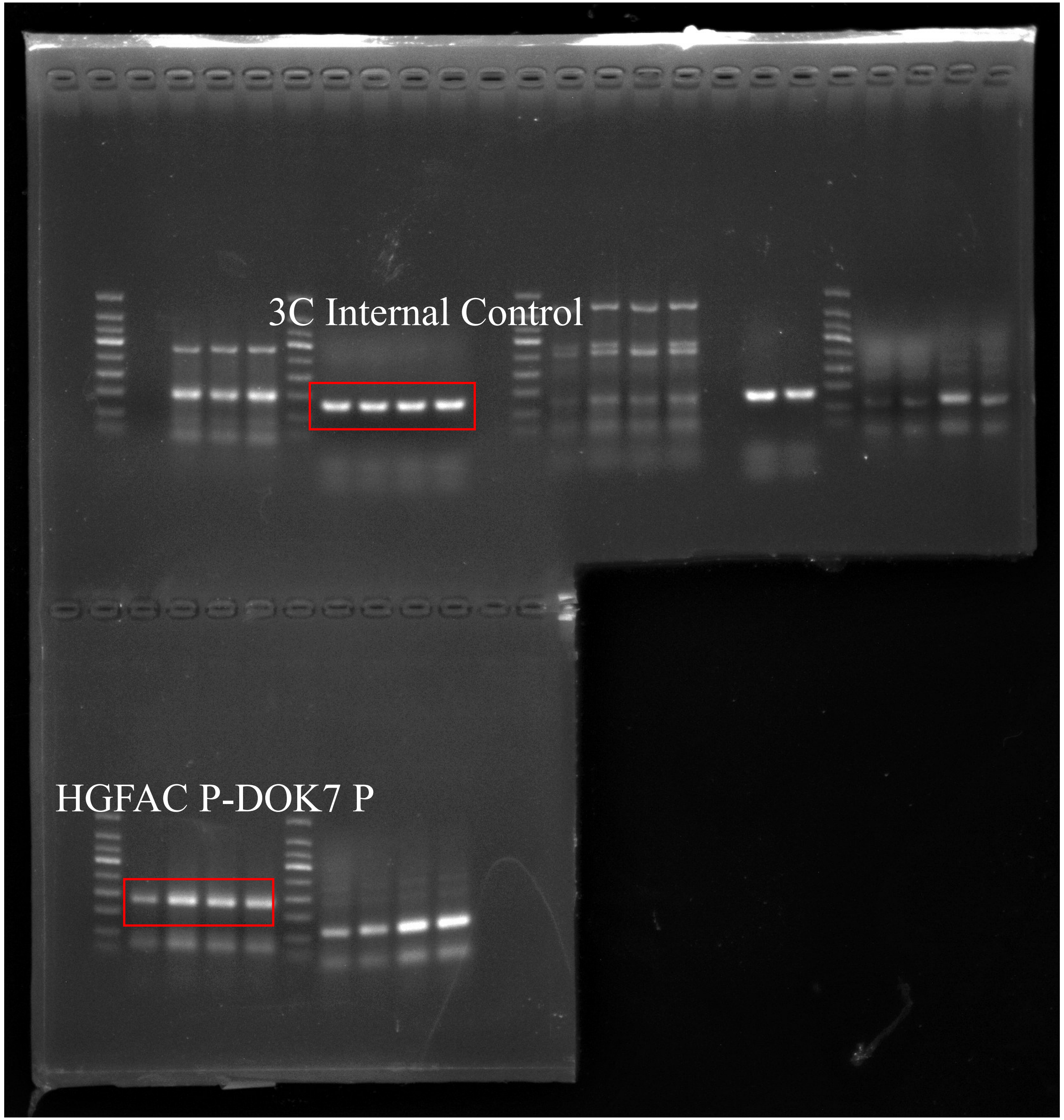

Figure S7A Original western blot image

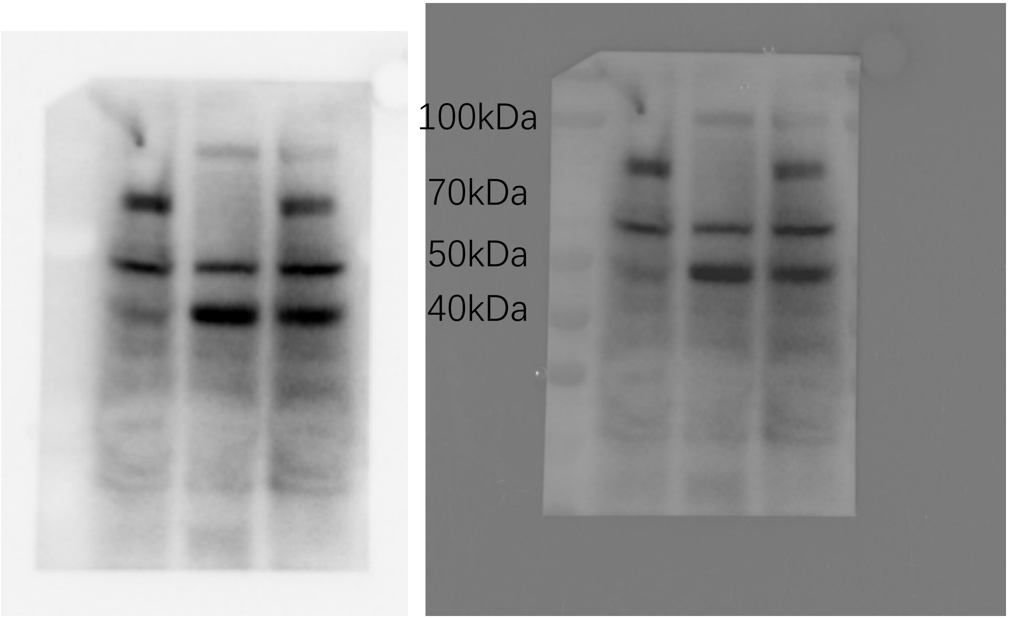

MECP2

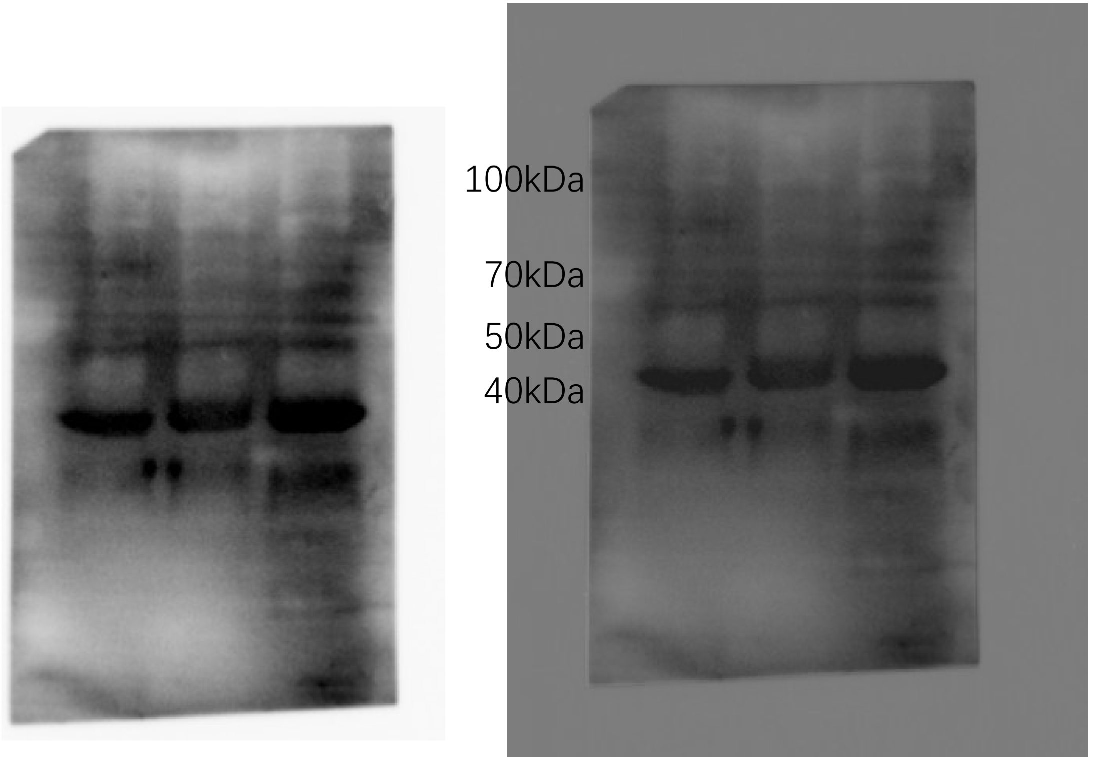

β-actin

Figure S7C Raw agarose gel electrophoresis image of ChIP-qPCR products

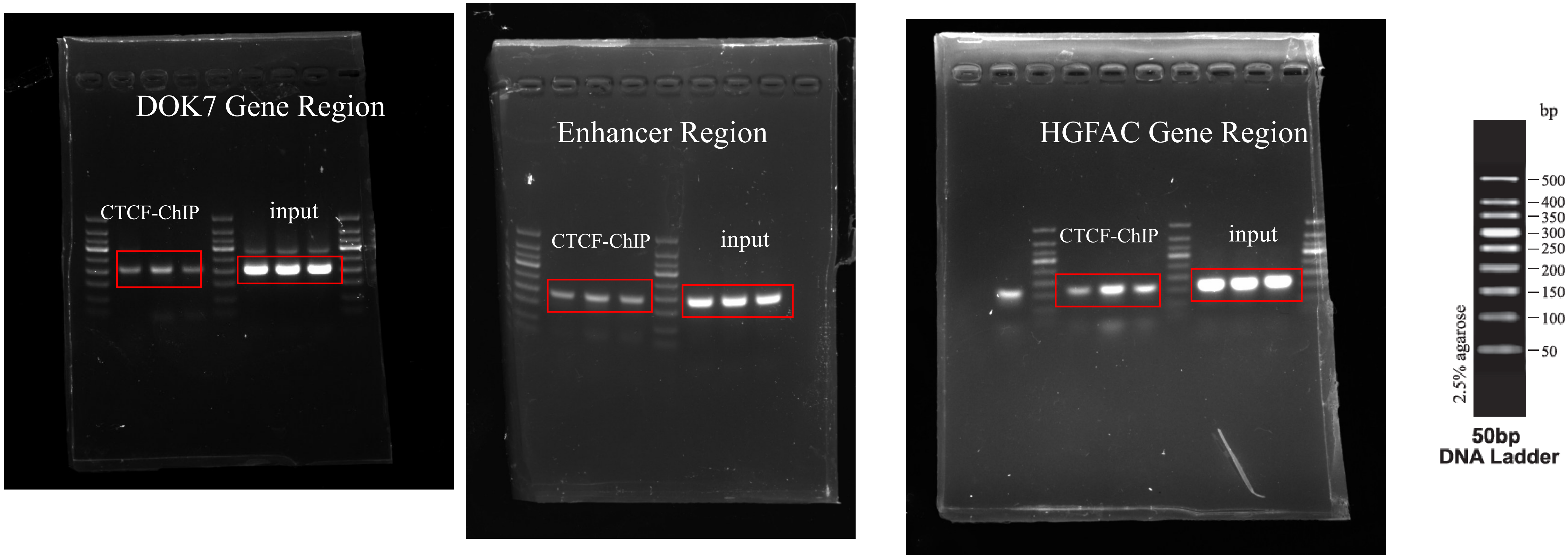

Figure S7D Raw agarose gel electrophoresis image of 3C products

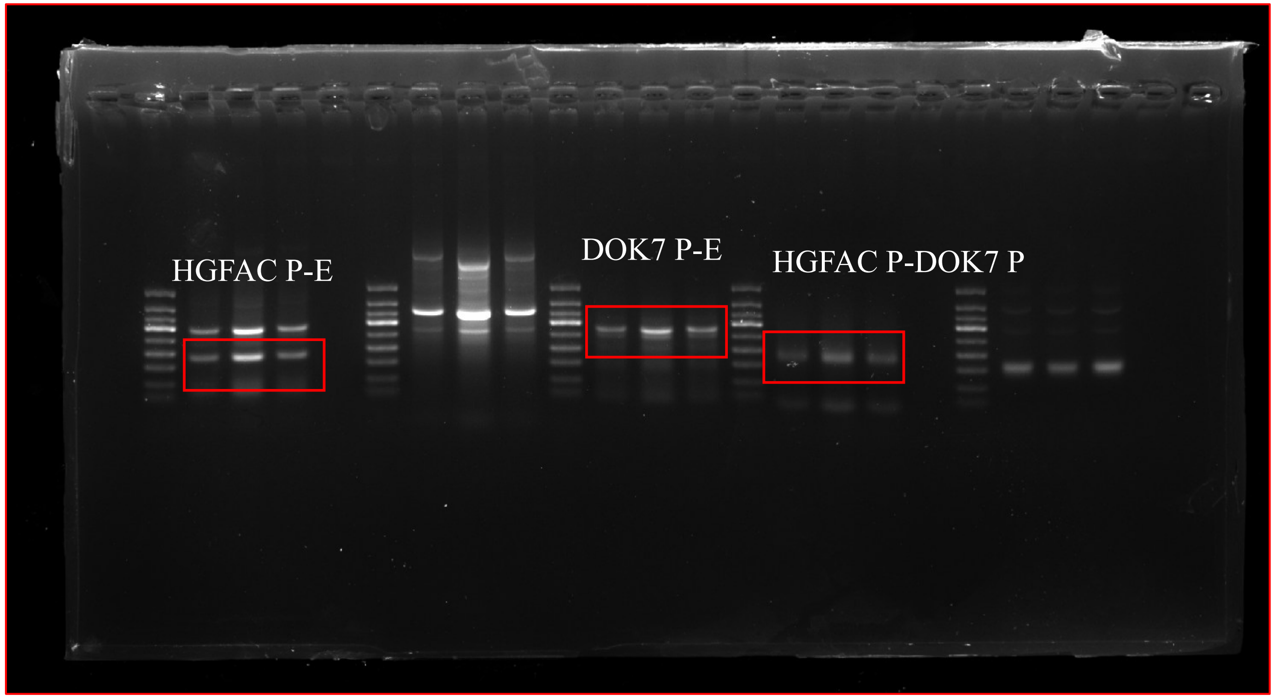

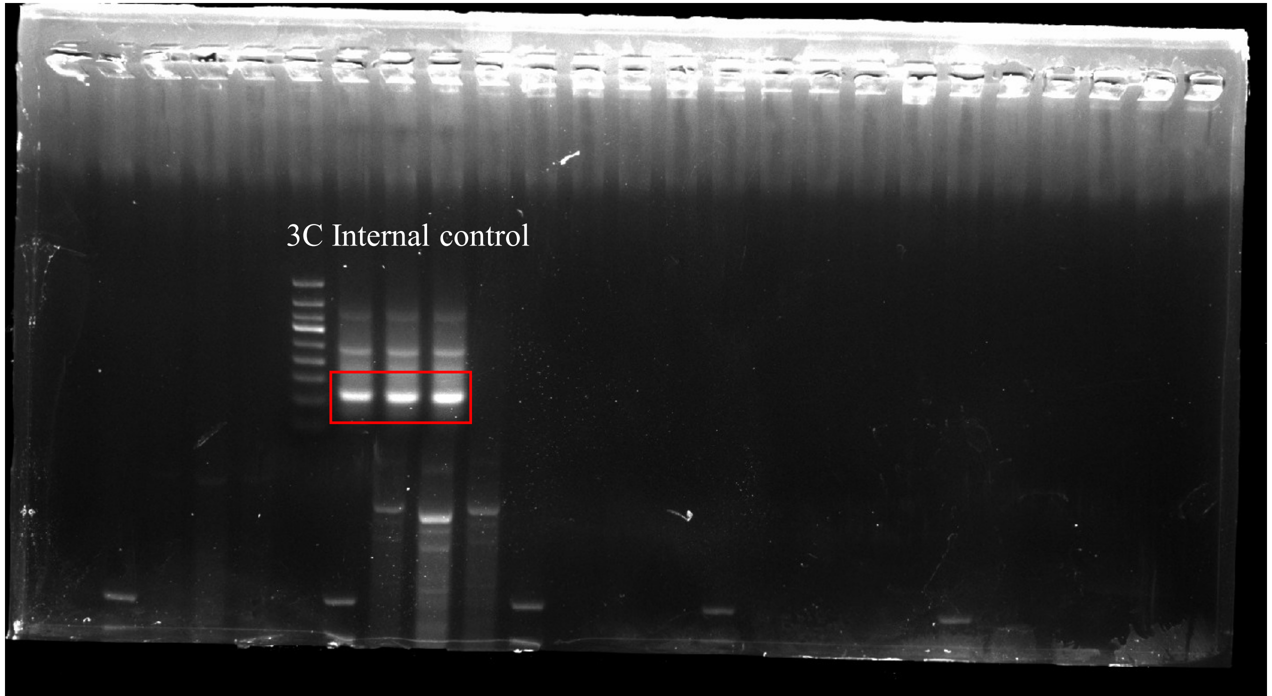

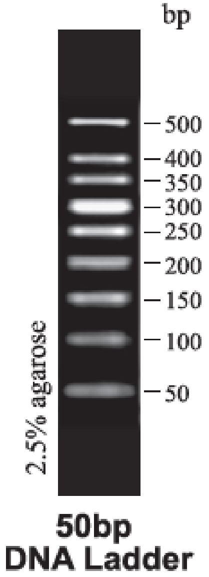

Supplement: Supplementary file 3 — original gel pictures [file 41419_2026_8837_MOESM3_ESM.pdf]
